# Highly pathogenic H5N6 avian influenza virus outbreak in *Pavo cristatus* in Jiangxi Province, China

**DOI:** 10.1080/22221751.2019.1586411

**Published:** 2019-03-11

**Authors:** Meng Li, Shengyong Feng, Sanfu Lv, Jing Luo, Jianli Guo, Jianhua Sun, Hongxuan He

**Affiliations:** a National Research Center for Wildlife Borne Diseases, Institute of Zoology, Chinese Academy of Sciences, Beijing, People’s Republic of China; b College of Life Sciences, University of the Chinese Academy of Sciences, Beijing, People’s Republic of China; c Center of Animal Disease Prevention and Control of Tong Zhou District, Beijing, People’s Republic of China

**Keywords:** Influenza virus, H5N6, Pavo Cristatus

## Abstract

Highly pathogenic avian influenza (HPAI) is a persistent threat to poultry, wild birds, humans, and other mammals. The continually evolving HPAI H5N6 virus has induced great losses in breeding industries in growing regions around the world. In this study, we confirmed an outbreak of the HPAI H5N6 virus in captive *Pavo cristatus* in Jiangxi Province, China. The causative agents H5N6 viruses were isolated and designated JS01, JS02, and K10. Animal experiments showed that all three isolates exhibited high pathogenicity to chickens, but they need adaption to effectively infect mice. A phylogenetic analysis showed that all three isolates were clustered in H5 clade 2.3.4.4c. No novel genetic reassortant was found in JS01, JS02, and K10 viruses. It was estimated that JS01, JS02, and K10 H5N6 viruses were direct descendants of the H5N6 virus circulating in South of China. The estimated divergence time from tMRCAs was anywhere between May 2014 to June 2016. Although the number of outbreaks of avian influenza decreased significantly in 2018, the threat from avian influenza to public health remains serious. Enhanced active surveillance is required to monitor the transmission and evolution of H5 influenza viruses.

To the editor,

Highly pathogenic avian influenza (HPAI) is a persistent threat to poultry, wild birds, humans, and other mammalian animals [1]. HPAI H5N6 virus is one of the major subtypes of the H5 clade 2.3.4.4 influenza viruses, and it induces great losses in breeding industries in growing regions around the world [2]. To date, approximately 1100 outbreaks of H5N6 AIV have been reported worldwide, including at least 23 human infection cases in China (FAO). Previous studies have reported that HPAI H5N6 influenza viruses are continuously evolving and have become the predominant H5 clade AIV detected in poultry in China. Furthermore, the H5N6 influenza virus has undergone reassortment with other type A influenza viruses (IAVs) to yield several other IAV subtypes (for example, H10N6 and H3N6) [3]. Therefore, the H5N6 influenza virus constitutes a serious threat to the poultry industry and human health.

On Jan 24, 2018, there was an outbreak of an acute infectious disease in *Pavo cristatus* at a breeding farm in Jiangxi Province, China. Approximately 86% *P. cristatus* were infected and died due to infection. The typical clinical signs include depression, fever, diarrhoea, and neural system disorder (Supplementary Video 1). All of the infected birds died within 1 d of the onset of clinical manifestations. Through necropsies, gastrointestinal tract necrosis, pulmonary lesions, and meningorrhagia were observed in the dead peafowls (Supplementary Figure 1). These results were confirmed by histopathological analysis (Supplementary Figure 2). We preliminarily speculated that the peafowls died due to avian influenza, Newcastle disease, or avian cholera. Neither the Newcastle disease virus nor *Pasteurella multocida* were detected. IAV-specific RT–PCR amplification yielded positive results. By inoculating 9-day-old embryonic eggs, three strains of the H5N6 virus were isolated, identified, and designated A/Pavo Cristatus/Jiangxi/JS01/2018 (JS01), A/ Pavo Cristatus/Jiangxi/JS02/2018 (JS02), and A/Pavo Cristatus/Jiangxi/K10/2018 (K10). No other subtype of influenza virus was detected or isolated. Based on the observation of clinical signs, histopathological analysis, RT–PCR detection, and virus isolation, we confirmed that the H5N6 influenza virus was the causative agent.

Whole genomes of the isolated viruses were sequenced and submitted to the NCBI database. Blast analysis suggested these isolated viruses exhibited the highest identity with other H5N6 viruses detected in Guangxi or Guangdong Province, China (Supplementary Table 1). To further understand the origin of these isolated viruses, we performed a phylogenetic analysis using MCC trees generated with BEAST (v1.8.4) software [4]. According to the resulting MCC trees, JS01, JS02, and K10 viruses were classified into H5 clade 2.3.4.4 (Figure 1). A previous report indicated that the H5N6 subtype influenza virus was a triple-reassortant virus that was composed of H5N1, H5N2, and H6N6 influenza viruses [5]. Our research provided consistent results. MCC trees derived from eight gene segments showed that JS01, JS02, and K10 viruses showed higher homology with viruses that were isolated from south China (Figure 1, Supplementary Figure 3, and Supplementary Table 2). This result suggests that the JS01, JS02, and K10 viruses were direct descendants of the H5N6 virus that caused endemics in South of China. The estimated divergence time from the most recent common ancestor (tMRCAs) was anywhere between May 2014 to Jun 2016 (Figure 1, Supplementary Figure 3, Supplementary Table 2). The evolutionary rates of the eight gene segments of these H5N6 viruses were estimated based on Bayesian analysis. The results showed that HA and NA genes had evolved faster than other analysed gene segments (Supplementary Table 3).

**Figure 1.**
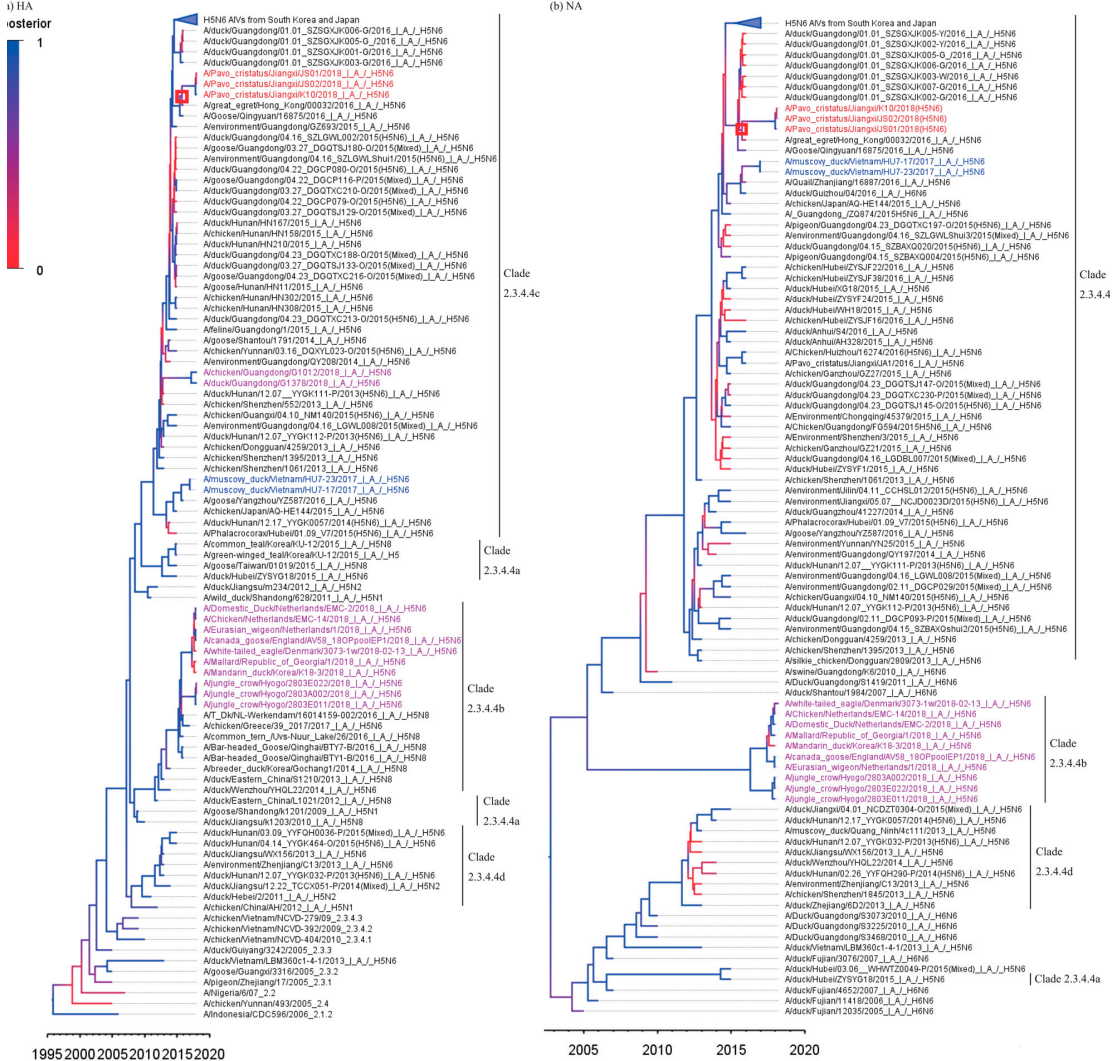
MCC trees of HA and NA genes of the JS01, JS02, and K10 viruses. The JS01, JS02, and K10 AIVs were classified as H5 Clade 2.3.4.4c viruses. The estimated divergent time of HA and NA genes of the JS01, JS02, and K10 viruses from their tMRCAs was Sep 2015. The trees were generated by BEAST software (V1.8.4) and edited by FigTree (v1.4.3). The red squares represent the tMRCAs of isolated viruses. The JS01, JS02, and K10 AIVs were marked by red text, Hu7-17 and Hu7-23 were marked by blue text, while other H5N6 AIVs isolated in 2018 were marked by purple text.

To understand their potential threat to public health, we further analysed the key amino acids in the viral proteins of JS01, JS02, and K10 viruses (Supplementary Table 4). First, we noted that JS01, JS02, and K10 possessed multiple basic amino acids (-RERRRKR-) at the HA cleavage site, which is a characteristic of highly pathogenic influenza viruses and indicates they are highly pathogenic for chickens [6]. Second, we noted that the receptor binding site (RBS) of HA1 retains the amino acid residues Q222 and G224 (H5 numbering) in the HA protein, which contributes to increased binding to Sia-2,3 avian-type receptors [7]. Third, we noted an 11-amino-acid deletion in the NA protein stalk region. This deletion was shown to regulate the replication, virulence and host tropism of IAVs [8]. Then we also observed residue L672 in the PA protein, H99 and I138 in the PB1 protein, and E627 and D701 in the PB2 protein; these residues confer low adaption to mammals and a limited capability for air-borne transmission [9]. Importantly, no drug-resistant mutations were seen in the M2 protein of the JS01, JS02, and K10 viruses.

Animal experiments showed that all three isolates were highly pathogenic to chickens (IVPI = 3.0). Additionally, a titre of 10^6^ PFU of isolated viruses cannot cause a lethal infection in 7-week-old BALB/c female mice.

In this study, we confirmed an outbreak of the HPAI H5N6 virus in captive *P. cristatus* in Jiangxi Province, China. The results showed that no novel genetic reassortant was found in JS01, JS02, and K10 viruses, which indicated that they are the direct descendant of H5N6 viruses circulating in south China. No waterfowls or migratory birds can be seen around the farm, so we hypothesized that domestic birds or resident birds are the infection vectors. Unfortunately, we did not found enough evidence to illustrate the source of the JS01, JS02, and K10 viruses. In 2018, approximately 760 outbreaks of avian influenza were reported worldwide (FAO), while the number of outbreaks in 2017 and 2016 were 3690 and 2608 respectively. The number of outbreaks has decreased significantly; however, the threat from avian influenza to poultry and public health remains serious. It is important to note that the H5N6 influenza virus is continuously evolving and spreading across different countries. For example, we observed that several H5N6 AIVs isolated from Europe, Korea, and Japan in 2017 or 2018 represented novel reassortant H5 clade 2.3.4.4b viruses composed of H5N6 AIVs, H5N8 AIVs, and LPAIVs (Supplementary Figure 3), which is consistent with previous reports [10,11]. We also noted that H5N6 AIVs two novel reassortant H5N6 viruses (A/Md/Vietnam/Hu7-17/2017 and A/Md/Vietnam/Hu7-23/2017) in Vietnam (Supplementary Figure 3). Based on the MCC trees, we found that Hu7-17 and Hu7-23 showed higher homology with H5N6 AIV derived from Japan (A/Ck/Japan/AQ-HE144/2015 (H5N6)). The results indicated the long-distance transmission of H5N6 AIVs from East Asia to South-east Asia by wild birds or trade (Supplementary Figure 3). H5 clade 2.3.4.4b AIV is a potential threat to South-east Asia and China. The overall results once again remind us that enhanced active surveillance is required to monitor the transmission and evolution of H5 influenza viruses.

## Supplementary Material

Supplemental Material
