# Supplementary material for: Highly pathogenic H5N6 avian influenza virus outbreak in Pavo cristatus in Jiangxi Province, China
Source: Emerg Microbes Infect. 2019 Mar 11;8(1):377–80. doi: 10.1080/22221751.2019.1586411 (PMC6455133; doi:10.1080/22221751.2019.1586411)
Supplement: Supplemental Material [file TEMI_A_1586411_SM9500.zip › Supplementary Material/Supplementary_Figure_1_to_Supplementary_Figure_3.pdf]

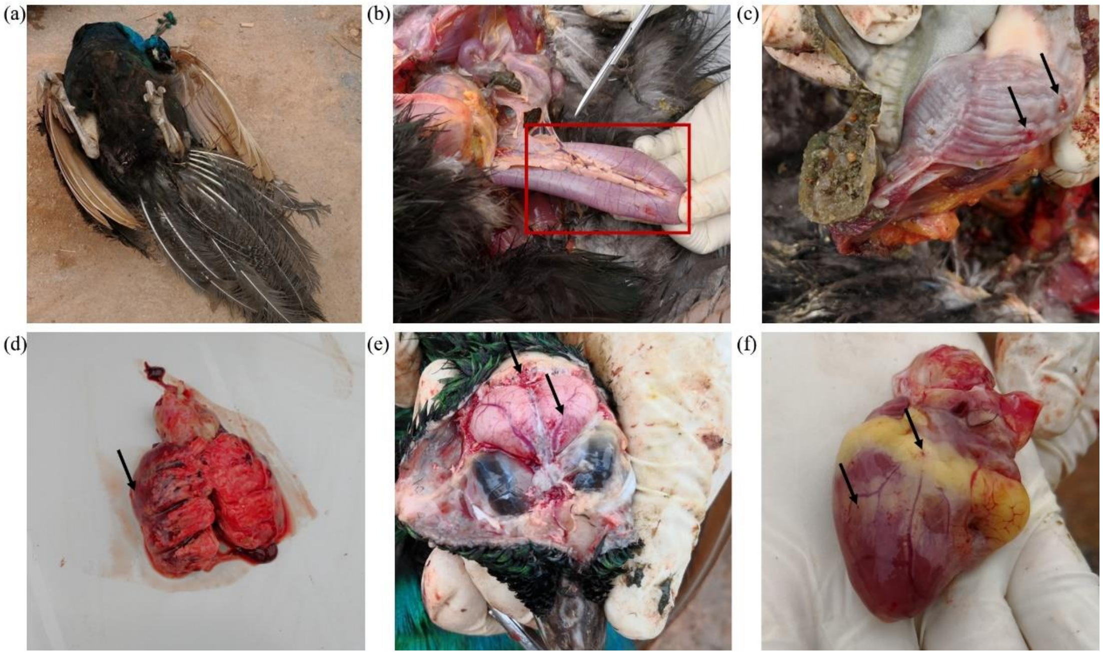

Supplementary figure 1 Pathogenicity of dead *Pavo Cristatus*. (a) The *Pavo Cristatus* dead due to highly contagious infectious disease. In addition, Intestine necrosis (b), stomach lesions (c), pulmonary hemorrhage (d), meningorrhagia (e), and heart hemorrhage were noted.

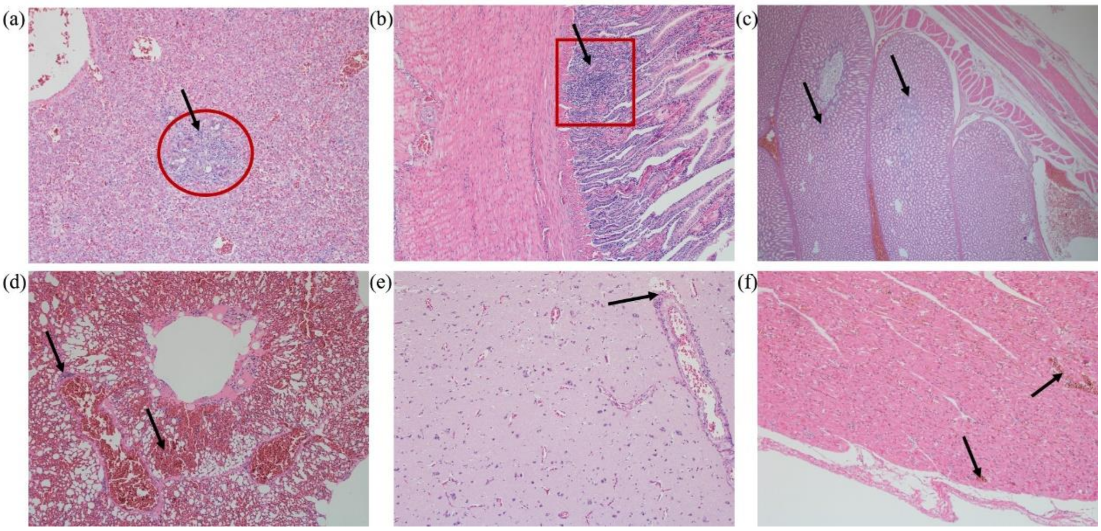

Supplementary figure 2 Histopathological analysis of dead *Pavo Cristatus*. Inflammatory cells infiltration were observed in liver (a), intestine (b), stomach (c), lung (d), and brain(e). In addition, erythrocyte diapedesis were noted in lung (d) and heart (f).

(a) PB2

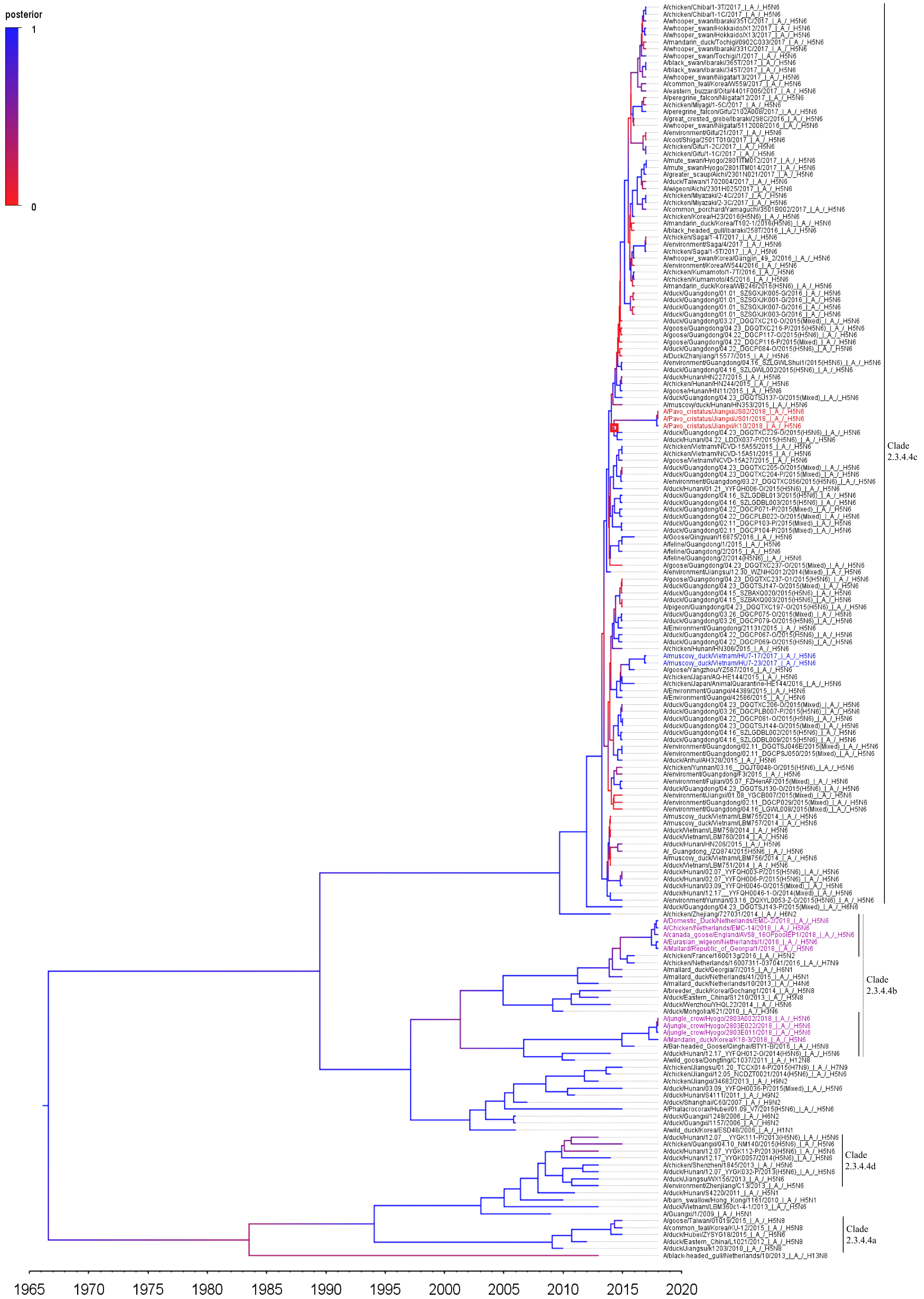
posterior
1
0
Clade 2.3.4.4c
Clade 2.3.4.4b
Clade 2.3.4.4d
Clade 2.3.4.4a
1965
1970
1975
1980
1985
1990
1995
2000
2005
2010
2015
2020

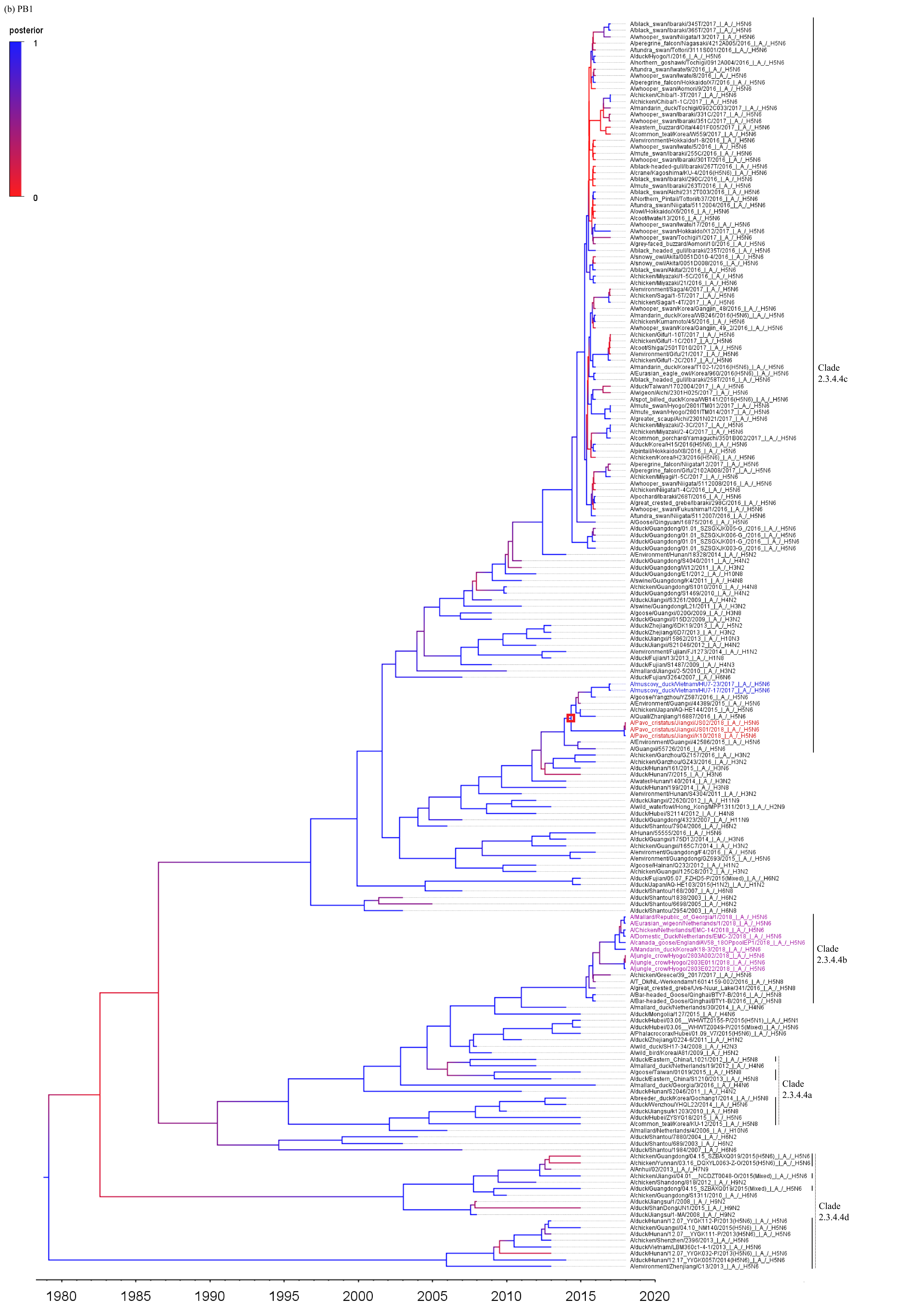

(b) PB1
posterior
1
0
Clade 2.3.4.4c
Clade 2.3.4.4b
Clade 2.3.4.4a
Clade 2.3.4.4d
1980
1985
1990
1995
2000
2005
2010
2015
2020

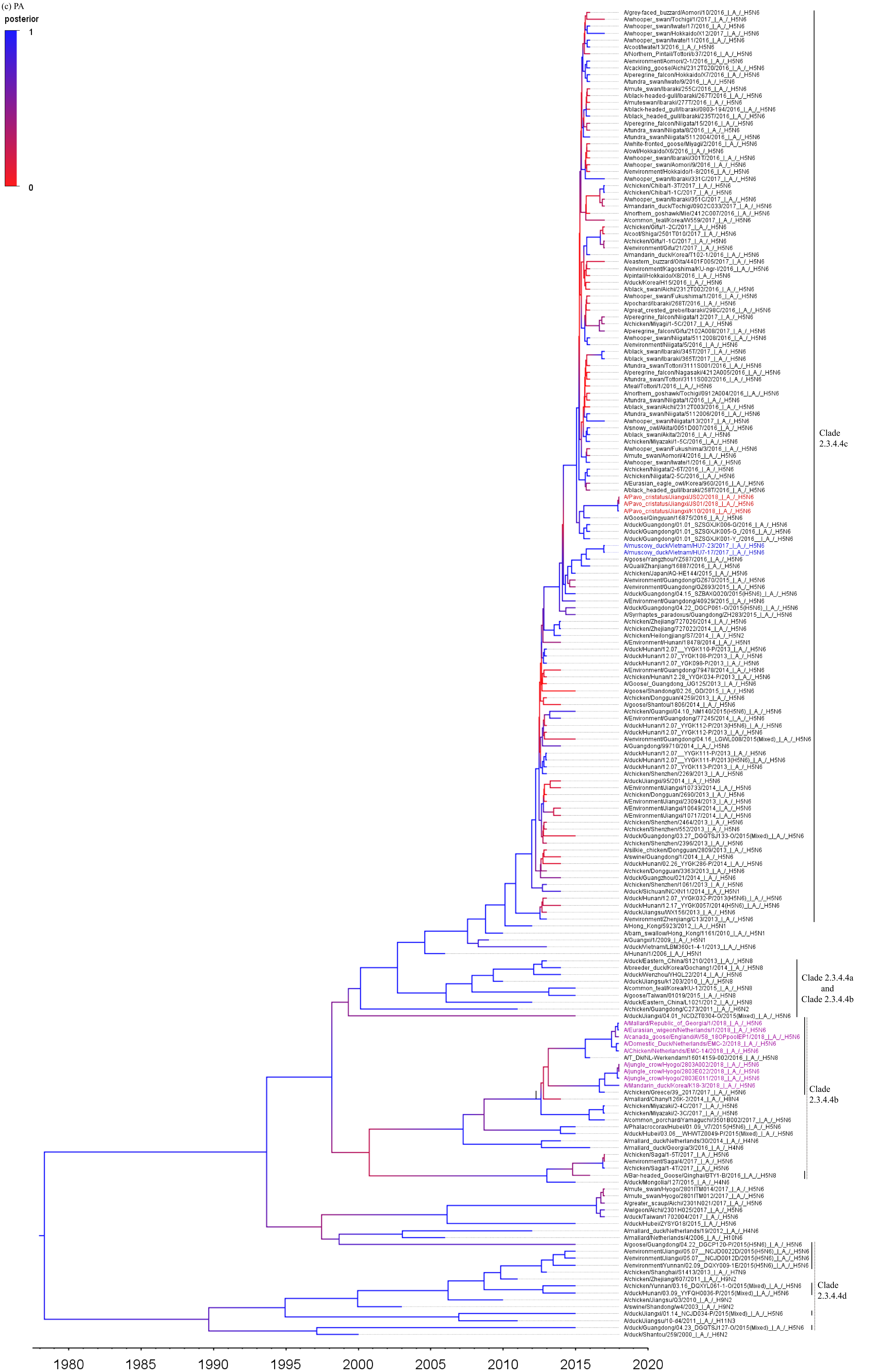
(c) PA
posterior
1
0
A/grey-faced_buzzard/Aomori/10/2016_|_A_/_H5N6
A/whooper_swan/Tochigi/1/2017_|_A_/_H5N6
A/whooper_swan/Iwate/17/2016_|_A_/_H5N6
A/whooper_swan/Hokkaido/X12/2017_|_A_/_H5N6
A/whooper_swan/Iwate/11/2016_|_A_/_H5N6
A/coot/Iwate/13/2016_|_A_/_H5N6
A/Northern_Pintail/Tottori/b37/2016_|_A_/_H5N6
A/environment/Aomori/2-1/2016_|_A_/_H5N6
A/cackling_goose/Aichi/2312T020/2016_|_A_/_H5N6
A/peregrine_falcon/Hokkaido/X7/2016_|_A_/_H5N6
A/tundra_swan/Iwate/9/2016_|_A_/_H5N6
A/mute_swan/Ibaraki/255C/2016_|_A_/_H5N6
A/black-headed-gull/Ibaraki/267T/2016_|_A_/_H5N6
A/muteswan/Ibaraki/277T/2016_|_A_/_H5N6
A/black-headed_gull/Ibaraki/0803-194/2016_|_A_/_H5N6
A/black_headed_gull/Ibaraki/235T/2016_|_A_/_H5N6
A/peregrine_falcon/Niigata/15/2016_|_A_/_H5N6
A/tundra_swan/Niigata/8/2016_|_A_/_H5N6
A/tundra_swan/Niigata/5112004/2016_|_A_/_H5N6
A/white-fronted_goose/Miyagi/2/2016_|_A_/_H5N6
A/owl/Hokkaido/X6/2016_|_A_/_H5N6
A/whooper_swan/Ibaraki/301T/2016_|_A_/_H5N6
A/whooper_swan/Aomori/9/2016_|_A_/_H5N6
A/environment/Hokkaido/1-8/2016_|_A_/_H5N6
A/whooper_swan/Ibaraki/331C/2017_|_A_/_H5N6
A/chicken/Chiba/1-3T/2017_|_A_/_H5N6
A/chicken/Chiba/1-1C/2017_|_A_/_H5N6
A/whooper_swan/Ibaraki/351C/2017_|_A_/_H5N6
A/mandarin_duck/Tochigi/0902C033/2017_|_A_/_H5N6
A/northern_goshawk/Mie/2412C007/2016_|_A_/_H5N6
A/common_teal/Korea/W559/2017_|_A_/_H5N6
A/chicken/Gifu/1-2C/2017_|_A_/_H5N6
A/coot/Shiga/2501T010/2017_|_A_/_H5N6
A/chicken/Gifu/1-1C/2017_|_A_/_H5N6
A/environment/Gifu/21/2017_|_A_/_H5N6
A/mandarin_duck/Korea/T102-1/2016_|_A_/_H5N6
A/eastern_buzzard/Oita/4401F005/2017_|_A_/_H5N6
A/environment/Kagoshima/KU-ngr-I/2016_|_A_/_H5N6
A/pintail/Hokkaido/X8/2016_|_A_/_H5N6
A/duck/Korea/H15/2016_|_A_/_H5N6
A/black_swan/Aichi/2312T002/2016_|_A_/_H5N6
A/whooper_swan/Fukushima/1/2016_|_A_/_H5N6
A/pochard/Ibaraki/268T/2016_|_A_/_H5N6
A/great_crested_grebe/Ibaraki/298C/2016_|_A_/_H5N6
A/peregrine_falcon/Niigata/12/2017_|_A_/_H5N6
A/chicken/Miyagi/1-5C/2017_|_A_/_H5N6
A/peregrine_falcon/Gifu/2102A008/2017_|_A_/_H5N6
A/whooper_swan/Niigata/5112008/2016_|_A_/_H5N6
A/environment/Niigata/5/2016_|_A_/_H5N6
A/black_swan/Ibaraki/345T/2017_|_A_/_H5N6
A/black_swan/Ibaraki/365T/2017_|_A_/_H5N6
A/tundra_swan/Tottori/3111S001/2016_|_A_/_H5N6
A/peregrine_falcon/Nagasaki/4212A005/2016_|_A_/_H5N6
A/tundra_swan/Tottori/3111S002/2016_|_A_/_H5N6
A/teal/Tottori/1/2016_|_A_/_H5N6
A/northern_goshawk/Tochigi/0912A004/2016_|_A_/_H5N6
A/tundra_swan/Niigata/1/2016_|_A_/_H5N6
A/black_swan/Aichi/2312T003/2016_|_A_/_H5N6
A/tundra_swan/Niigata/5112006/2016_|_A_/_H5N6
A/whooper_swan/Niigata/13/2017_|_A_/_H5N6
A/snowy_owl/Akita/0051D007/2016_|_A_/_H5N6
A/black_swan/Akita/2/2016_|_A_/_H5N6
A/chicken/Miyazaki/1-5C/2016_|_A_/_H5N6
A/whooper_swan/Fukushima/3/2016_|_A_/_H5N6
A/mute_swan/Aomori/4/2016_|_A_/_H5N6
A/whooper_swan/Iwate/1/2016_|_A_/_H5N6
A/chicken/Niigata/2-6T/2016_|_A_/_H5N6
A/chicken/Niigata/2-5C/2016_|_A_/_H5N6
A/Eurasian_eagle_owl/Korea/960/2016_|_A_/_H5N6
A/black_headed_gull/Ibaraki/258T/2016_|_A_/_H5N6
A/Pavo_cristatus/Jiangxi/JS02/2018_|_A_/_H5N6
A/Pavo_cristatus/Jiangxi/JS01/2018_|_A_/_H5N6
A/Pavo_cristatus/Jiangxi/K10/2018_|_A_/_H5N6
A/Goose/Qingyuan/16875/2016_|_A_/_H5N6
A/duck/Guangdong/01.01_SZSGXJK006-G/2016_|_A_/_H5N6
A/duck/Guangdong/01.01_SZSGXJK005-G_/2016_|_A_/_H5N6
A/duck/Guangdong/01.01_SZSGXJK001-Y_/2016__|_A_/_H5N6
A/muscovy_duck/Vietnam/HU7-23/2017_|_A_/_H5N6
A/muscovy_duck/Vietnam/HU7-17/2017_|_A_/_H5N6
A/goose/Yangzhou/YZ587/2016_|_A_/_H5N6
A/Quail/Zhanjiang/16887/2016_|_A_/_H5N6
A/chicken/Japan/AQ-HE144/2015_|_A_/_H5N6
A/environment/Guangdong/GZ670/2015_|_A_/_H5N6
A/environment/Guangdong/GZ693/2015_|_A_/_H5N6
A/duck/Guangdong/04.15_SZBAXQ020/2015(H5N6)_|_A_/_H5N6
A/Environment/Guangdong/40929/2015_|_A_/_H5N6
A/duck/Guangdong/04.22_DGCP061-O/2015(H5N6)_|_A_/_H5N6
A/Syrrhaptes_paradoxus/Guangdong/ZH283/2015_|_A_/_H5N6
A/chicken/Zhejiang/727026/2014_|_A_/_H5N6
A/chicken/Zhejiang/727022/2014_|_A_/_H5N6
A/chicken/Heilongjiang/S7/2014_|_A_/_H5N2
A/Environment/Hunan/18478/2014_|_A_/_H5N1
A/duck/Hunan/12.07__YYGK110-P/2013_|_A_/_H5N6
A/duck/Hunan/12.07_YYGK108-P/2013_|_A_/_H5N6
A/duck/Hunan/12.07_YGK098-P/2013_|_A_/_H5N6
A/Environment/Guangdong/79478/2014_|_A_/_H5N6
A/chicken/Hunan/12.28_YYGK034-P/2013_|_A_/_H5N6
A/Goose/_Guangdong_/JG125/2013_|_A_/_H5N6
A/goose/Shandong/02.26_GD/2015_|_A_/_H5N6
A/chicken/Dongguan/4259/2013_|_A_/_H5N6
A/goose/Shantou/1806/2014_|_A_/_H5N6
A/chicken/Guangxi/04.10_NM140/2015(H5N6)_|_A_/_H5N6
A/Environment/Guangdong/77245/2014_|_A_/_H5N6
A/duck/Hunan/12.07_YYGK112-P/2013(H5N6)_|_A_/_H5N6
A/duck/Hunan/12.07_YYGK112-P/2013_|_A_/_H5N6
A/environment/Guangdong/04.16_LGWL008/2015(Mixed)_|_A_/_H5N6
A/Guangdong/99710/2014_|_A_/_H5N6
A/duck/Hunan/12.07__YYGK111-P/2013_|_A_/_H5N6
A/duck/Hunan/12.07__YYGK111-P/2013(H5N6)_|_A_/_H5N6
A/duck/Hunan/12.07_YYGK113-P/2013_|_A_/_H5N6
A/chicken/Shenzhen/2269/2013_|_A_/_H5N6
A/duck/Jiangxi/95/2014_|_A_/_H5N6
A/Environment/Jiangxi/10733/2014_|_A_/_H5N6
A/chicken/Dongguan/2690/2013_|_A_/_H5N6
A/Environment/Jiangxi/23094/2013_|_A_/_H5N6
A/Environment/Jiangxi/10649/2014_|_A_/_H5N6
A/Environment/Jiangxi/10717/2014_|_A_/_H5N6
A/chicken/Shenzhen/2464/2013_|_A_/_H5N6
A/chicken/Shenzhen/552/2013_|_A_/_H5N6
A/duck/Guangdong/03.27_DGQTSJ133-O/2015(Mixed)_|_A_/_H5N6
A/chicken/Shenzhen/2396/2013_|_A_/_H5N6
A/silkie_chicken/Dongguan/2809/2013_|_A_/_H5N6
A/swine/Guangdong/1/2014_|_A_/_H5N6
A/duck/Hunan/02.26_YYGK286-P/2014_|_A_/_H5N6
A/chicken/Dongguan/3363/2013_|_A_/_H5N6
A/duck/Guangzhou/021/2014_|_A_/_H5N6
A/chicken/Shenzhen/1061/2013_|_A_/_H5N6
A/duck/Sichuan/NCXN11/2014_|_A_/_H5N1
A/duck/Hunan/12.07_YYGK032-P/2013(H5N6)_|_A_/_H5N6
A/duck/Hunan/12.17_YYGK0057/2014(H5N6)_|_A_/_H5N6
A/duck/Jiangsu/WX156/2013_|_A_/_H5N6
A/environment/Zhenjiang/C13/2013_|_A_/_H5N6
Clade 2.3.4.4c
A/Hong_Kong/5923/2012_|_A_/_H5N1
A/barn_swallow/Hong_Kong/1161/2010_|_A_/_H5N1
A/Guangxi/1/2009_|_A_/_H5N1
A/duck/Vietnam/LBM360c1-4-1/2013_|_A_/_H5N6
A/Hunan/1/2006_|_A_/_H5N1
A/duck/Eastern_China/S1210/2013_|_A_/_H5N8
A/breeder_duck/Korea/Gochang1/2014_|_A_/_H5N8
A/duck/Wenzhou/YHQL22/2014_|_A_/_H5N6
A/duck/Jiangsu/k1203/2010_|_A_/_H5N8
A/common_teal/Korea/KU-12/2015_|_A_/_H5N8
A/goose/Taiwan/01019/2015_|_A_/_H5N8
A/duck/Eastern_China/L1021/2012_|_A_/_H5N8
A/chicken/Guangdong/C273/2011_|_A_/_H6N2
A/duck/Jiangxi/04.01_NCDZT0304-O/2015(Mixed)_|_A_/_H5N6
Clade 2.3.4.4a and Clade 2.3.4.4b
A/Mallard/Republic_of_Georgia/1/2018_|_A_/_H5N6
A/Eurasian_wigeon/Netherlands/1/2018_|_A_/_H5N6
A/canada_goose/England/AV58_18OPpoolEP1/2018_|_A_/_H5N6
A/Domestic_Duck/Netherlands/EMC-2/2018_|_A_/_H5N6
A/Chicken/Netherlands/EMC-14/2018_|_A_/_H5N6
A/T_Dk/NL-Werkendam/16014159-002/2016_|_A_/_H5N8
A/jungle_crow/Hyogo/2803A002/2018_|_A_/_H5N6
A/jungle_crow/Hyogo/2803E022/2018_|_A_/_H5N6
A/jungle_crow/Hyogo/2803E011/2018_|_A_/_H5N6
A/Mandarin_duck/Korea/K18-3/2018_|_A_/_H5N6
A/chicken/Greece/39_2017/2017_|_A_/_H5N6
A/mallard/Chany/126K-2/2014_|_A_/_H8N4
A/chicken/Miyazaki/2-4C/2017_|_A_/_H5N6
A/chicken/Miyazaki/2-3C/2017_|_A_/_H5N6
A/common_porchard/Yamaguchi/3501B002/2017_|_A_/_H5N6
A/Phalacrocorax/Hubei/01.09_V7/2015(H5N6)_|_A_/_H5N6
A/duck/Hubei/03.06__WHWTZ0049-P/2015(Mixed)_|_A_/_H5N6
A/mallard_duck/Netherlands/30/2014_|_A_/_H4N6
A/mallard_duck/Georgia/3/2016_|_A_/_H4N6
A/chicken/Saga/1-5T/2017_|_A_/_H5N6
A/environment/Saga/4/2017_|_A_/_H5N6
A/chicken/Saga/1-4T/2017_|_A_/_H5N6
A/Bar-headed_Goose/Qinghai/BTY1-B/2016_|_A_/_H5N8
Clade 2.3.4.4b
A/duck/Mongolia/127/2015_|_A_/_H4N6
A/mute_swan/Hyogo/2801ITM014/2017_|_A_/_H5N6
A/mute_swan/Hyogo/2801ITM012/2017_|_A_/_H5N6
A/greater_scaup/Aichi/2301N021/2017_|_A_/_H5N6
A/wigeon/Aichi/2301H025/2017_|_A_/_H5N6
A/duck/Taiwan/1702004/2017_|_A_/_H5N6
A/duck/Hubei/ZYSYG18/2015_|_A_/_H5N6
A/mallard_duck/Netherlands/19/2012_|_A_/_H4N6
A/mallard/Netherlands/4/2006_|_A_/_H10N6
A/goose/Guangdong/04.22_DGCP120-P/2015(H5N6)_|_A_/_H5N6
A/environment/Jiangxi/05.07__NCJD0022D/2015(H5N6)_|_A_/_H5N6
A/environment/Jiangxi/05.07__NCJD0012D/2015(H5N6)_|_A_/_H5N6
A/environment/Yunnan/02.09_DQXY009-1E/2015(H5N6)_|_A_/_H5N6
A/chicken/Shanghai/S1413/2013_|_A_/_H7N9
A/chicken/Zhejiang/607/2011_|_A_/_H9N2
A/chicken/Yunnan/03.16_DQXYL061-1-O/2015(Mixed)_|_A_/_H5N6
A/duck/Hunan/03.09_YYFQH0036-P/2015(Mixed)_|_A_/_H5N6
A/chicken/Jiangsu/Q3/2010_|_A_/_H9N2
A/swine/Shandong/w4/2003_|_A_/_H9N2
A/duck/Jiangxi/01.14_NCJD034-P/2015(Mixed)_|_A_/_H5N6
A/duck/Jiangsu/10-d4/2011_|_A_/_H11N3
A/duck/Guangdong/04.23_DGQTSJ127-O/2015(Mixed)_|_A_/_H5N6
A/duck/Shantou/259/2000_|_A_/_H6N2
Clade 2.3.4.4d
1980
1985
1990
1995
2000
2005
2010
2015
2020

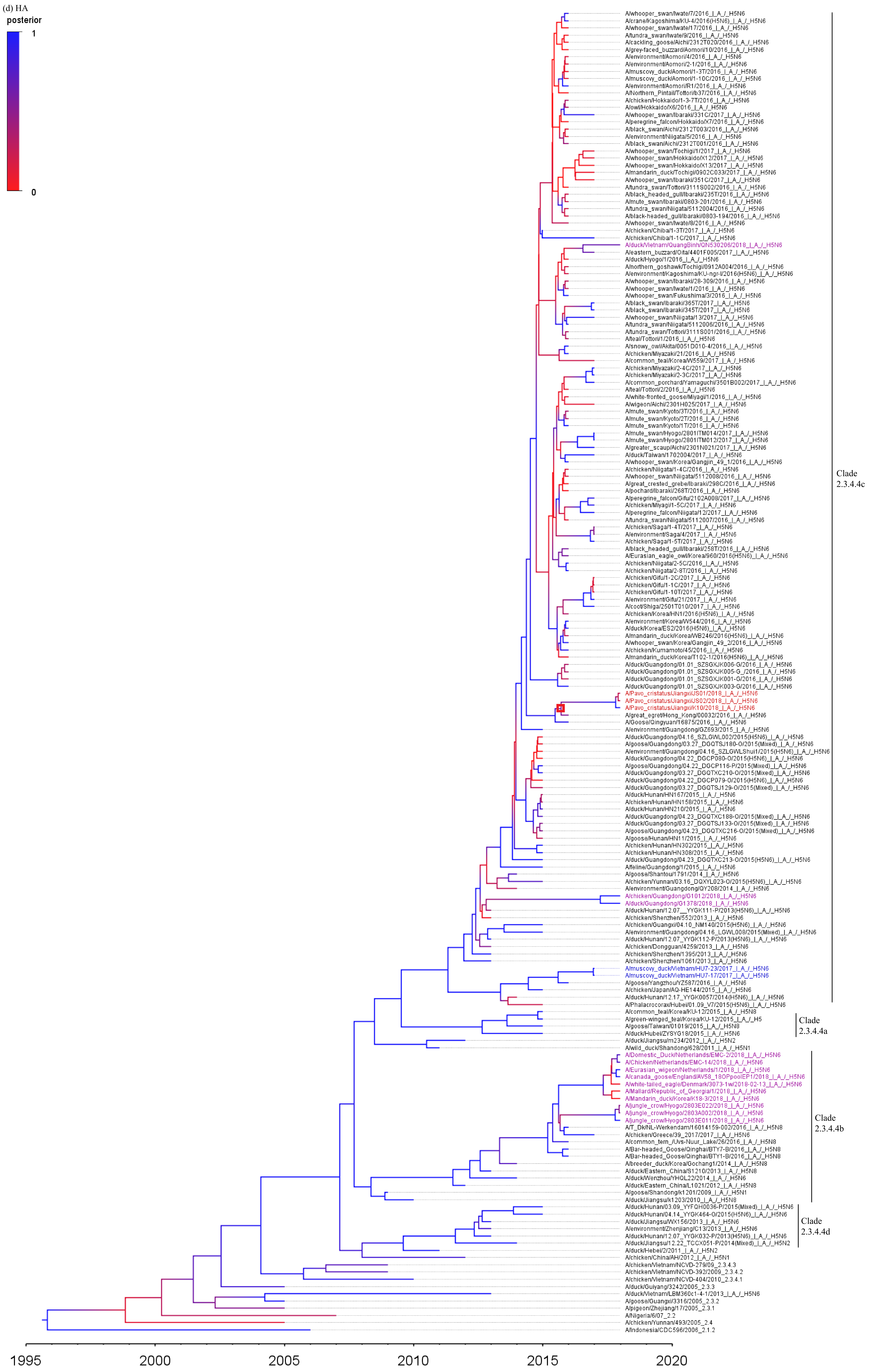

(d) HA
posterior
1
0
A/whooper_swan/Iwate/7/2016_|_A_/_H5N6
A/crane/Kagoshima/KU-4/2016(H5N6)_|_A_/_H5N6
A/whooper_swan/Iwate/17/2016_|_A_/_H5N6
A/tundra_swan/Iwate/9/2016_|_A_/_H5N6
A/cackling_goose/Aichi/2312T020/2016_|_A_/_H5N6
A/grey-faced_buzzard/Aomori/10/2016_|_A_/_H5N6
A/environment/Aomori/4/2016_|_A_/_H5N6
A/environment/Aomori/2-1/2016_|_A_/_H5N6
A/muscovy_duck/Aomori/1-3T/2016_|_A_/_H5N6
A/muscovy_duck/Aomori/1-10C/2016_|_A_/_H5N6
A/environment/Aomori/R1/2016_|_A_/_H5N6
A/Northern_Pintail/Tottori/b37/2016_|_A_/_H5N6
A/chicken/Hokkaido/1-3-7T/2016_|_A_/_H5N6
A/owl/Hokkaido/X6/2016_|_A_/_H5N6
A/whooper_swan/Ibaraki/331C/2017_|_A_/_H5N6
A/peregrine_falcon/Hokkaido/X7/2016_|_A_/_H5N6
A/black_swan/Aichi/2312T003/2016_|_A_/_H5N6
A/environment/Niigata/5/2016_|_A_/_H5N6
A/black_swan/Aichi/2312T001/2016_|_A_/_H5N6
A/whooper_swan/Tochigi/1/2017_|_A_/_H5N6
A/whooper_swan/Hokkaido/X12/2017_|_A_/_H5N6
A/whooper_swan/Hokkaido/X13/2017_|_A_/_H5N6
A/mandarin_duck/Tochigi/0902C033/2017_|_A_/_H5N6
A/whooper_swan/Ibaraki/351C/2017_|_A_/_H5N6
A/tundra_swan/Tottori/3111S002/2016_|_A_/_H5N6
A/black_headed_gull/Ibaraki/235T/2016_|_A_/_H5N6
A/mute_swan/Ibaraki/0803-201/2016_|_A_/_H5N6
A/tundra_swan/Niigata/5112004/2016_|_A_/_H5N6
A/black-headed_gull/Ibaraki/0803-194/2016_|_A_/_H5N6
A/whooper_swan/Iwate/8/2016_|_A_/_H5N6
A/chicken/Chiba/1-3T/2017_|_A_/_H5N6
A/chicken/Chiba/1-1C/2017_|_A_/_H5N6
A/duck/Vietnam/QuangBinh/QN530206/2018_|_A_/_H5N6
A/eastern_buzzard/Oita/4401F005/2017_|_A_/_H5N6
A/duck/Hyogo/1/2016_|_A_/_H5N6
A/northern_goshawk/Tochigi/0912A004/2016_|_A_/_H5N6
A/environment/Kagoshima/KU-ngr-I/2016(H5N6)_|_A_/_H5N6
A/whooper_swan/Ibaraki/28-309/2016_|_A_/_H5N6
A/whooper_swan/Iwate/1/2016_|_A_/_H5N6
A/whooper_swan/Fukushima/3/2016_|_A_/_H5N6
A/black_swan/Ibaraki/365T/2017_|_A_/_H5N6
A/black_swan/Ibaraki/345T/2017_|_A_/_H5N6
A/whooper_swan/Niigata/13/2017_|_A_/_H5N6
A/tundra_swan/Niigata/5112006/2016_|_A_/_H5N6
A/tundra_swan/Tottori/3111S001/2016_|_A_/_H5N6
A/teal/Tottori/1/2016_|_A_/_H5N6
A/snowy_owl/Akita/0051D010-4/2016_|_A_/_H5N6
A/chicken/Miyazaki/21/2016_|_A_/_H5N6
A/common_teal/Korea/W559/2017_|_A_/_H5N6
A/chicken/Miyazaki/2-4C/2017_|_A_/_H5N6
A/chicken/Miyazaki/2-3C/2017_|_A_/_H5N6
A/common_porchard/Yamaguchi/3501B002/2017_|_A_/_H5N6
A/teal/Tottori/2/2016_|_A_/_H5N6
A/white-fronted_goose/Miyagi/1/2016_|_A_/_H5N6
A/wigeon/Aichi/2301H025/2017_|_A_/_H5N6
A/mute_swan/Kyoto/3T/2016_|_A_/_H5N6
A/mute_swan/Kyoto/2T/2016_|_A_/_H5N6
A/mute_swan/Kyoto/1T/2016_|_A_/_H5N6
A/mute_swan/Hyogo/2801ITM014/2017_|_A_/_H5N6
A/mute_swan/Hyogo/2801ITM012/2017_|_A_/_H5N6
A/greater_scaup/Aichi/2301N021/2017_|_A_/_H5N6
A/duck/Taiwan/1702004/2017_|_A_/_H5N6
A/whooper_swan/Korea/Gangjin_49_1/2016_|_A_/_H5N6
A/chicken/Niigata/1-4C/2016_|_A_/_H5N6
A/whooper_swan/Niigata/5112008/2016_|_A_/_H5N6
A/great_crested_grebe/Ibaraki/298C/2016_|_A_/_H5N6
A/pochard/Ibaraki/268T/2016_|_A_/_H5N6
A/peregrine_falcon/Gifu/2102A008/2017_|_A_/_H5N6
A/chicken/Miyagi/1-5C/2017_|_A_/_H5N6
A/peregrine_falcon/Niigata/12/2017_|_A_/_H5N6
A/tundra_swan/Niigata/5112007/2016_|_A_/_H5N6
A/chicken/Saga/1-4T/2017_|_A_/_H5N6
A/environment/Saga/4/2017_|_A_/_H5N6
A/chicken/Saga/1-5T/2017_|_A_/_H5N6
A/black_headed_gull/Ibaraki/258T/2016_|_A_/_H5N6
A/Eurasian_eagle_owl/Korea/960/2016(H5N6)_|_A_/_H5N6
A/chicken/Niigata/2-5C/2016_|_A_/_H5N6
A/chicken/Niigata/2-8T/2016_|_A_/_H5N6
A/chicken/Gifu/1-2C/2017_|_A_/_H5N6
A/chicken/Gifu/1-1C/2017_|_A_/_H5N6
A/chicken/Gifu/1-10T/2017_|_A_/_H5N6
A/environment/Gifu/21/2017_|_A_/_H5N6
A/coot/Shiga/2501T010/2017_|_A_/_H5N6
A/chicken/Korea/HN1/2016(H5N6)_|_A_/_H5N6
A/environment/Korea/W544/2016_|_A_/_H5N6
A/duck/Korea/ES2/2016(H5N6)_|_A_/_H5N6
A/mandarin_duck/Korea/WB246/2016(H5N6)_|_A_/_H5N6
A/whooper_swan/Korea/Gangjin_49_2/2016_|_A_/_H5N6
A/chicken/Kumamoto/45/2016_|_A_/_H5N6
A/mandarin_duck/Korea/T102-1/2016(H5N6)_|_A_/_H5N6
A/duck/Guangdong/01.01_SZSGXJK006-G/2016_|_A_/_H5N6
A/duck/Guangdong/01.01_SZSGXJK005-G_/2016_|_A_/_H5N6
A/duck/Guangdong/01.01_SZSGXJK001-G/2016_|_A_/_H5N6
A/duck/Guangdong/01.01_SZSGXJK003-G/2016_|_A_/_H5N6
A/Pavo_cristatus/Jiangxi/JS01/2018_|_A_/_H5N6
A/Pavo_cristatus/Jiangxi/JS02/2018_|_A_/_H5N6
A/Pavo_cristatus/Jiangxi/K10/2018_|_A_/_H5N6
A/great_egret/Hong_Kong/00032/2016_|_A_/_H5N6
A/Goose/Qingyuan/16875/2016_|_A_/_H5N6
A/environment/Guangdong/GZ693/2015_|_A_/_H5N6
A/duck/Guangdong/04.16_SZLGWL002/2015(H5N6)_|_A_/_H5N6
A/goose/Guangdong/03.27_DGQTSJ180-O/2015(Mixed)_|_A_/_H5N6
A/environment/Guangdong/04.16_SZLGWLShui1/2015(H5N6)_|_A_/_H5N6
A/duck/Guangdong/04.22_DGCP080-O/2015(H5N6)_|_A_/_H5N6
A/goose/Guangdong/04.22_DGCP116-P/2015(Mixed)_|_A_/_H5N6
A/duck/Guangdong/03.27_DGQTXC210-O/2015(Mixed)_|_A_/_H5N6
A/duck/Guangdong/04.22_DGCP079-O/2015(H5N6)_|_A_/_H5N6
A/duck/Guangdong/03.27_DGQTSJ129-O/2015(Mixed)_|_A_/_H5N6
A/duck/Hunan/HN167/2015_|_A_/_H5N6
A/chicken/Hunan/HN158/2015_|_A_/_H5N6
A/duck/Hunan/HN210/2015_|_A_/_H5N6
A/duck/Guangdong/04.23_DGQTXC188-O/2015(Mixed)_|_A_/_H5N6
A/duck/Guangdong/03.27_DGQTSJ133-O/2015(Mixed)_|_A_/_H5N6
A/goose/Guangdong/04.23_DGQTXC216-O/2015(Mixed)_|_A_/_H5N6
A/goose/Hunan/HN11/2015_|_A_/_H5N6
A/chicken/Hunan/HN302/2015_|_A_/_H5N6
A/chicken/Hunan/HN308/2015_|_A_/_H5N6
A/duck/Guangdong/04.23_DGQTXC213-O/2015(H5N6)_|_A_/_H5N6
A/feline/Guangdong/1/2015_|_A_/_H5N6
A/goose/Shantou/1791/2014_|_A_/_H5N6
A/chicken/Yunnan/03.16_DQXYL023-O/2015(H5N6)_|_A_/_H5N6
A/environment/Guangdong/QY208/2014_|_A_/_H5N6
A/chicken/Guangdong/G1012/2018_|_A_/_H5N6
A/duck/Guangdong/G1378/2018_|_A_/_H5N6
A/duck/Hunan/12.07__YYGK111-P/2013(H5N6)_|_A_/_H5N6
A/chicken/Shenzhen/552/2013_|_A_/_H5N6
A/chicken/Guangxi/04.10_NM140/2015(H5N6)_|_A_/_H5N6
A/environment/Guangdong/04.16_LGWL008/2015(Mixed)_|_A_/_H5N6
A/duck/Hunan/12.07_YYGK112-P/2013(H5N6)_|_A_/_H5N6
A/chicken/Dongguan/4259/2013_|_A_/_H5N6
A/chicken/Shenzhen/1395/2013_|_A_/_H5N6
A/chicken/Shenzhen/1061/2013_|_A_/_H5N6
A/muscovy_duck/Vietnam/HU7-23/2017_|_A_/_H5N6
A/muscovy_duck/Vietnam/HU7-17/2017_|_A_/_H5N6
A/goose/Yangzhou/YZ587/2016_|_A_/_H5N6
A/chicken/Japan/AQ-HE144/2015_|_A_/_H5N6
A/duck/Hunan/12.17_YYGK0057/2014(H5N6)_|_A_/_H5N6
A/Phalacrocorax/Hubei/01.09_V7/2015(H5N6)_|_A_/_H5N6
A/common_teal/Korea/KU-12/2015_|_A_/_H5N8
A/green-winged_teal/Korea/KU-12/2015_|_A_/_H5
A/goose/Taiwan/01019/2015_|_A_/_H5N8
A/duck/Hubei/ZYSYG18/2015_|_A_/_H5N6
A/duck/Jiangsu/m234/2012_|_A_/_H5N2
A/wild_duck/Shandong/628/2011_|_A_/_H5N1
A/Domestic_Duck/Netherlands/EMC-2/2018_|_A_/_H5N6
A/Chicken/Netherlands/EMC-14/2018_|_A_/_H5N6
A/Eurasian_wigeon/Netherlands/1/2018_|_A_/_H5N6
A/canada_goose/England/AV58_18OPpoolEP1/2018_|_A_/_H5N6
A/white-tailed_eagle/Denmark/3073-1w/2018-02-13_|_A_/_H5N6
A/Mallard/Republic_of_Georgia/1/2018_|_A_/_H5N6
A/Mandarin_duck/Korea/K18-3/2018_|_A_/_H5N6
A/jungle_crow/Hyogo/2803E022/2018_|_A_/_H5N6
A/jungle_crow/Hyogo/2803A002/2018_|_A_/_H5N6
A/jungle_crow/Hyogo/2803E011/2018_|_A_/_H5N6
A/T_Dk/NL-Werkendam/16014159-002/2016_|_A_/_H5N8
A/chicken/Greece/39_2017/2017_|_A_/_H5N6
A/common_tern_/Uvs-Nuur_Lake/26/2016_|_A_/_H5N8
A/Bar-headed_Goose/Qinghai/BTY7-B/2016_|_A_/_H5N8
A/Bar-headed_Goose/Qinghai/BTY1-B/2016_|_A_/_H5N8
A/breeder_duck/Korea/Gochang1/2014_|_A_/_H5N8
A/duck/Eastern_China/S1210/2013_|_A_/_H5N8
A/duck/Wenzhou/YHQL22/2014_|_A_/_H5N6
A/duck/Eastern_China/L1021/2012_|_A_/_H5N8
A/goose/Shandong/k1201/2009_|_A_/_H5N1
A/duck/Jiangsu/k1203/2010_|_A_/_H5N8
A/duck/Hunan/03.09_YYFQH0036-P/2015(Mixed)_|_A_/_H5N6
A/duck/Hunan/04.14_YYGK464-O/2015(H5N6)_|_A_/_H5N6
A/duck/Jiangsu/WX156/2013_|_A_/_H5N6
A/environment/Zhenjiang/C13/2013_|_A_/_H5N6
A/duck/Hunan/12.07_YYGK032-P/2013(H5N6)_|_A_/_H5N6
A/duck/Jiangsu/12.22_TCCX051-P/2014(Mixed)_|_A_/_H5N2
A/duck/Hebei/2/2011_|_A_/_H5N2
A/chicken/China/AH/2012_|_A_/_H5N1
A/chicken/Vietnam/NCVD-279/09_2.3.4.3
A/chicken/Vietnam/NCVD-392/2009_2.3.4.2
A/chicken/Vietnam/NCVD-404/2010_2.3.4.1
A/duck/Guiyang/3242/2005_2.3.3
A/duck/Vietnam/LBM360c1-4-1/2013_|_A_/_H5N6
A/goose/Guangxi/3316/2005_2.3.2
A/pigeon/Zhejiang/17/2005_2.3.1
A/Nigeria/6/07_2.2
A/chicken/Yunnan/493/2005_2.4
A/Indonesia/CDC596/2006_2.1.2
Clade 2.3.4.4c
Clade 2.3.4.4a
Clade 2.3.4.4b
Clade 2.3.4.4d
1995
2000
2005
2010
2015
2020

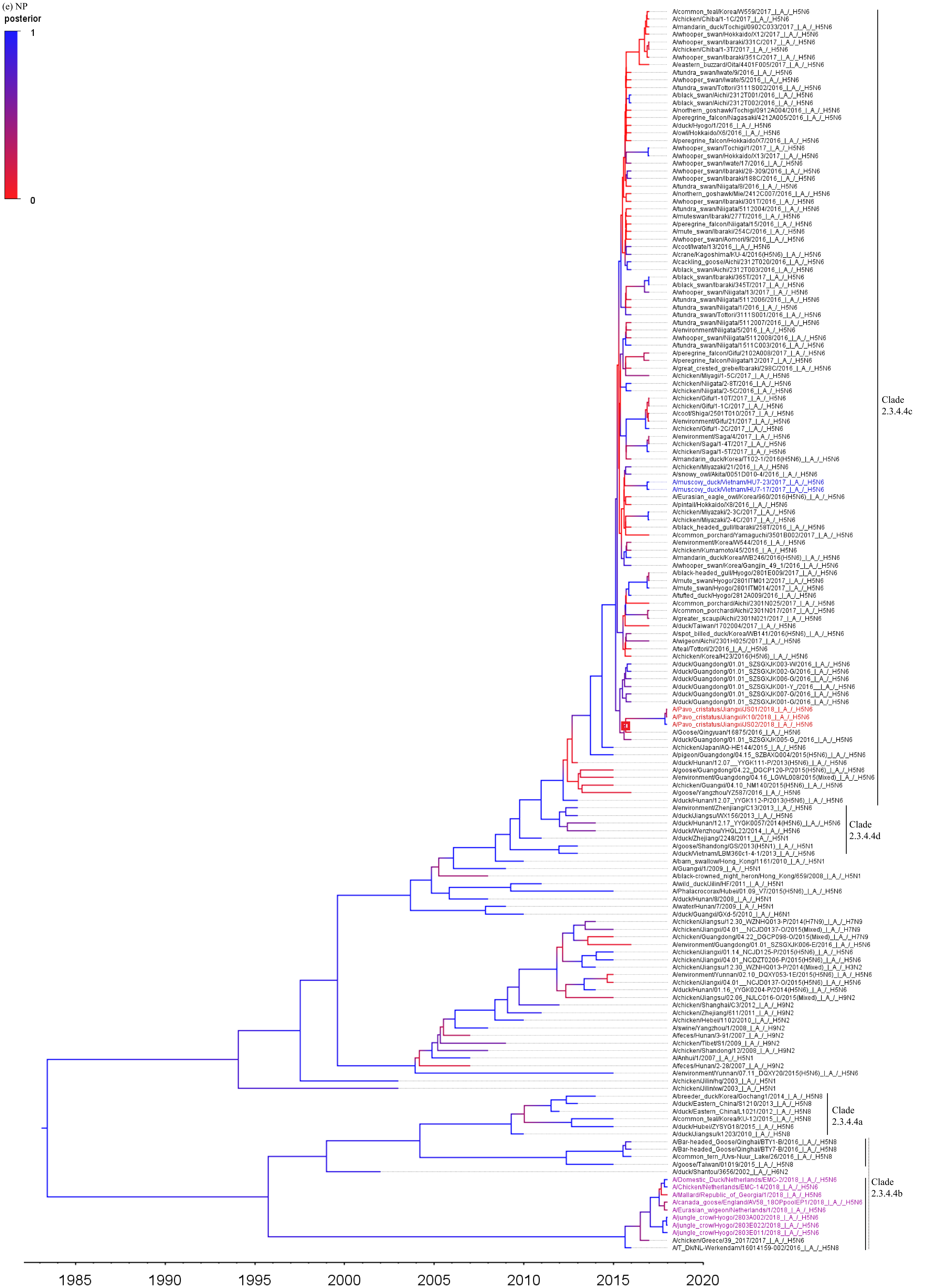

(e) NP
posterior
1
0
Clade 2.3.4.4c
Clade 2.3.4.4d
Clade 2.3.4.4a
Clade 2.3.4.4b
1985
1990
1995
2000
2005
2010
2015
2020

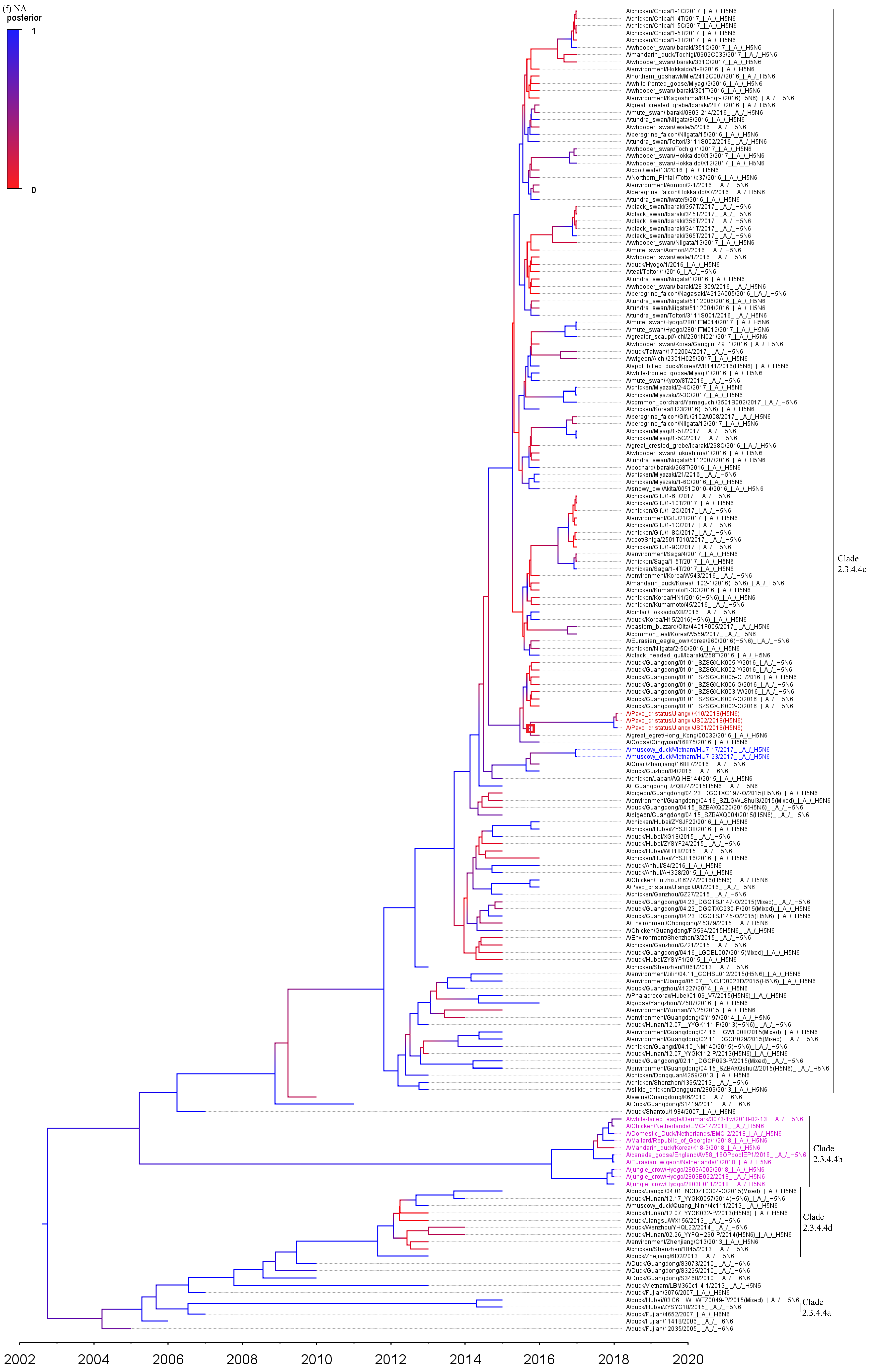
(f) NA
posterior
1
0
A/chicken/Chiba/1-1C/2017_|_A_/_H5N6
A/chicken/Chiba/1-4T/2017_|_A_/_H5N6
A/chicken/Chiba/1-5C/2017_|_A_/_H5N6
A/chicken/Chiba/1-5T/2017_|_A_/_H5N6
A/chicken/Chiba/1-3T/2017_|_A_/_H5N6
A/whooper_swan/Ibaraki/351C/2017_|_A_/_H5N6
A/mandarin_duck/Tochigi/0902C033/2017_|_A_/_H5N6
A/whooper_swan/Ibaraki/331C/2017_|_A_/_H5N6
A/environment/Hokkaido/1-8/2016_|_A_/_H5N6
A/northern_goshawk/Mie/2412C007/2016_|_A_/_H5N6
A/white-fronted_goose/Miyagi/2/2016_|_A_/_H5N6
A/whooper_swan/Ibaraki/301T/2016_|_A_/_H5N6
A/environment/Kagoshima/KU-ngr-I/2016(H5N6)_|_A_/_H5N6
A/great_crested_grebe/Ibaraki/287T/2016_|_A_/_H5N6
A/mute_swan/Ibaraki/0803-214/2016_|_A_/_H5N6
A/tundra_swan/Niigata/8/2016_|_A_/_H5N6
A/whooper_swan/Iwate/5/2016_|_A_/_H5N6
A/peregrine_falcon/Niigata/15/2016_|_A_/_H5N6
A/tundra_swan/Tottori/3111S002/2016_|_A_/_H5N6
A/whooper_swan/Tochigi/1/2017_|_A_/_H5N6
A/whooper_swan/Hokkaido/X13/2017_|_A_/_H5N6
A/whooper_swan/Hokkaido/X12/2017_|_A_/_H5N6
A/coot/Iwate/13/2016_|_A_/_H5N6
A/Northern_Pintail/Tottori/b37/2016_|_A_/_H5N6
A/environment/Aomori/2-1/2016_|_A_/_H5N6
A/peregrine_falcon/Hokkaido/X7/2016_|_A_/_H5N6
A/tundra_swan/Iwate/9/2016_|_A_/_H5N6
A/black_swan/Ibaraki/357T/2017_|_A_/_H5N6
A/black_swan/Ibaraki/345T/2017_|_A_/_H5N6
A/black_swan/Ibaraki/356T/2017_|_A_/_H5N6
A/black_swan/Ibaraki/341T/2017_|_A_/_H5N6
A/black_swan/Ibaraki/365T/2017_|_A_/_H5N6
A/whooper_swan/Niigata/13/2017_|_A_/_H5N6
A/mute_swan/Aomori/4/2016_|_A_/_H5N6
A/whooper_swan/Iwate/1/2016_|_A_/_H5N6
A/duck/Hyogo/1/2016_|_A_/_H5N6
A/teal/Tottori/1/2016_|_A_/_H5N6
A/tundra_swan/Niigata/1/2016_|_A_/_H5N6
A/whooper_swan/Ibaraki/28-309/2016_|_A_/_H5N6
A/peregrine_falcon/Nagasaki/4212A005/2016_|_A_/_H5N6
A/tundra_swan/Niigata/5112006/2016_|_A_/_H5N6
A/tundra_swan/Niigata/5112004/2016_|_A_/_H5N6
A/tundra_swan/Tottori/3111S001/2016_|_A_/_H5N6
A/mute_swan/Hyogo/2801ITM014/2017_|_A_/_H5N6
A/mute_swan/Hyogo/2801ITM012/2017_|_A_/_H5N6
A/greater_scaup/Aichi/2301N021/2017_|_A_/_H5N6
A/whooper_swan/Korea/Gangjin_49_1/2016_|_A_/_H5N6
A/duck/Taiwan/1702004/2017_|_A_/_H5N6
A/wigeon/Aichi/2301H025/2017_|_A_/_H5N6
A/spot_billed_duck/Korea/WB141/2016(H5N6)_|_A_/_H5N6
A/white-fronted_goose/Miyagi/1/2016_|_A_/_H5N6
A/mute_swan/Kyoto/8T/2016_|_A_/_H5N6
A/chicken/Miyazaki/2-4C/2017_|_A_/_H5N6
A/chicken/Miyazaki/2-3C/2017_|_A_/_H5N6
A/common_porchard/Yamaguchi/3501B002/2017_|_A_/_H5N6
A/chicken/Korea/H23/2016(H5N6)_|_A_/_H5N6
A/peregrine_falcon/Gifu/2102A008/2017_|_A_/_H5N6
A/peregrine_falcon/Niigata/12/2017_|_A_/_H5N6
A/chicken/Miyagi/1-5T/2017_|_A_/_H5N6
A/chicken/Miyagi/1-5C/2017_|_A_/_H5N6
A/great_crested_grebe/Ibaraki/298C/2016_|_A_/_H5N6
A/whooper_swan/Fukushima/1/2016_|_A_/_H5N6
A/tundra_swan/Niigata/5112007/2016_|_A_/_H5N6
A/pochard/Ibaraki/268T/2016_|_A_/_H5N6
A/chicken/Miyazaki/21/2016_|_A_/_H5N6
A/chicken/Miyazaki/1-6C/2016_|_A_/_H5N6
A/snowy_owl/Akita/0051D010-4/2016_|_A_/_H5N6
A/chicken/Gifu/1-6T/2017_|_A_/_H5N6
A/chicken/Gifu/1-10T/2017_|_A_/_H5N6
A/chicken/Gifu/1-2C/2017_|_A_/_H5N6
A/environment/Gifu/21/2017_|_A_/_H5N6
A/chicken/Gifu/1-1C/2017_|_A_/_H5N6
A/chicken/Gifu/1-8C/2017_|_A_/_H5N6
A/coot/Shiga/2501T010/2017_|_A_/_H5N6
A/chicken/Gifu/1-9C/2017_|_A_/_H5N6
A/environment/Saga/4/2017_|_A_/_H5N6
A/chicken/Saga/1-5T/2017_|_A_/_H5N6
A/chicken/Saga/1-4T/2017_|_A_/_H5N6
A/environment/Korea/W543/2016_|_A_/_H5N6
A/mandarin_duck/Korea/T102-1/2016(H5N6)_|_A_/_H5N6
A/chicken/Kumamoto/1-3C/2016_|_A_/_H5N6
A/chicken/Korea/HN1/2016(H5N6)_|_A_/_H5N6
A/chicken/Kumamoto/45/2016_|_A_/_H5N6
A/pintail/Hokkaido/X8/2016_|_A_/_H5N6
A/duck/Korea/H15/2016(H5N6)_|_A_/_H5N6
A/eastern_buzzard/Oita/4401F005/2017_|_A_/_H5N6
A/common_teal/Korea/W559/2017_|_A_/_H5N6
A/Eurasian_eagle_owl/Korea/960/2016(H5N6)_|_A_/_H5N6
A/chicken/Niigata/2-5C/2016_|_A_/_H5N6
A/black_headed_gull/Ibaraki/258T/2016_|_A_/_H5N6
A/duck/Guangdong/01.01_SZSGXJK005-Y/2016_|_A_/_H5N6
A/duck/Guangdong/01.01_SZSGXJK002-Y/2016_|_A_/_H5N6
A/duck/Guangdong/01.01_SZSGXJK005-G_/2016_|_A_/_H5N6
A/duck/Guangdong/01.01_SZSGXJK006-G/2016_|_A_/_H5N6
A/duck/Guangdong/01.01_SZSGXJK003-W/2016_|_A_/_H5N6
A/duck/Guangdong/01.01_SZSGXJK007-G/2016_|_A_/_H5N6
A/duck/Guangdong/01.01_SZSGXJK002-G/2016_|_A_/_H5N6
A/Pavo_cristatus/Jiangxi/K10/2018(H5N6)
A/Pavo_cristatus/Jiangxi/JS02/2018(H5N6)
A/Pavo_cristatus/Jiangxi/JS01/2018(H5N6)
A/great_egret/Hong_Kong/00032/2016_|_A_/_H5N6
A/Goose/Qingyuan/16875/2016_|_A_/_H5N6
A/muscovy_duck/Vietnam/HU7-17/2017_|_A_/_H5N6
A/muscovy_duck/Vietnam/HU7-23/2017_|_A_/_H5N6
A/Quail/Zhanjiang/16887/2016_|_A_/_H5N6
A/duck/Guizhou/04/2016_|_A_/_H6N6
A/chicken/Japan/AQ-HE144/2015_|_A_/_H5N6
A/_Guangdong_/ZQ874/2015H5N6_|_A_/_H5N6
A/pigeon/Guangdong/04.23_DGQTXC197-O/2015(H5N6)_|_A_/_H5N6
A/environment/Guangdong/04.16_SZLGWLShui3/2015(Mixed)_|_A_/_H5N6
A/duck/Guangdong/04.15_SZBAXQ020/2015(H5N6)_|_A_/_H5N6
A/pigeon/Guangdong/04.15_SZBAXQ004/2015(H5N6)_|_A_/_H5N6
A/chicken/Hubei/ZYSJF22/2016_|_A_/_H5N6
A/chicken/Hubei/ZYSJF38/2016_|_A_/_H5N6
A/duck/Hubei/XG18/2015_|_A_/_H5N6
A/duck/Hubei/ZYSYF24/2015_|_A_/_H5N6
A/duck/Hubei/WH18/2015_|_A_/_H5N6
A/chicken/Hubei/ZYSJF16/2016_|_A_/_H5N6
A/duck/Anhui/S4/2016_|_A_/_H5N6
A/duck/Anhui/AH328/2015_|_A_/_H5N6
A/Chicken/Huizhou/16274/2016(H5N6)_|_A_/_H5N6
A/Pavo_cristatus/Jiangxi/JA1/2016_|_A_/_H5N6
A/chicken/Ganzhou/GZ27/2015_|_A_/_H5N6
A/duck/Guangdong/04.23_DGQTSJ147-O/2015(Mixed)_|_A_/_H5N6
A/duck/Guangdong/04.23_DGQTXC230-P/2015(Mixed)_|_A_/_H5N6
A/duck/Guangdong/04.23_DGQTSJ145-O/2015(H5N6)_|_A_/_H5N6
A/Environment/Chongqing/45379/2015_|_A_/_H5N6
A/Chicken/Guangdong/FG594/2015H5N6_|_A_/_H5N6
A/Environment/Shenzhen/3/2015_|_A_/_H5N6
A/chicken/Ganzhou/GZ21/2015_|_A_/_H5N6
A/duck/Guangdong/04.16_LGDBL007/2015(Mixed)_|_A_/_H5N6
A/duck/Hubei/ZYSYF1/2015_|_A_/_H5N6
A/chicken/Shenzhen/1061/2013_|_A_/_H5N6
A/environment/Jilin/04.11_CCHSL012/2015(H5N6)_|_A_/_H5N6
A/environment/Jiangxi/05.07__NCJD0023D/2015(H5N6)_|_A_/_H5N6
A/duck/Guangzhou/41227/2014_|_A_/_H5N6
A/Phalacrocorax/Hubei/01.09_V7/2015(H5N6)_|_A_/_H5N6
A/goose/Yangzhou/YZ587/2016_|_A_/_H5N6
A/environment/Yunnan/YN25/2015_|_A_/_H5N6
A/environment/Guangdong/QY197/2014_|_A_/_H5N6
A/duck/Hunan/12.07__YYGK111-P/2013(H5N6)_|_A_/_H5N6
A/environment/Guangdong/04.16_LGWL008/2015(Mixed)_|_A_/_H5N6
A/environment/Guangdong/02.11_DGCP029/2015(Mixed)_|_A_/_H5N6
A/chicken/Guangxi/04.10_NM140/2015(H5N6)_|_A_/_H5N6
A/duck/Hunan/12.07_YYGK112-P/2013(H5N6)_|_A_/_H5N6
A/duck/Guangdong/02.11_DGCP093-P/2015(Mixed)_|_A_/_H5N6
A/environment/Guangdong/04.15_SZBAXQshui2/2015(H5N6)_|_A_/_H5N6
A/chicken/Dongguan/4259/2013_|_A_/_H5N6
A/chicken/Shenzhen/1395/2013_|_A_/_H5N6
A/silkie_chicken/Dongguan/2809/2013_|_A_/_H5N6
Clade 2.3.4.4c
A/swine/Guangdong/K6/2010_|_A_/_H6N6
A/Duck/Guangdong/S1419/2011_|_A_/_H6N6
A/duck/Shantou/1984/2007_|_A_/_H6N6
A/white-tailed_eagle/Denmark/3073-1w/2018-02-13_|_A_/_H5N6
A/Chicken/Netherlands/EMC-14/2018_|_A_/_H5N6
A/Domestic_Duck/Netherlands/EMC-2/2018_|_A_/_H5N6
A/Mallard/Republic_of_Georgia/1/2018_|_A_/_H5N6
A/Mandarin_duck/Korea/K18-3/2018_|_A_/_H5N6
A/canada_goose/England/AV58_18OPpoolEP1/2018_|_A_/_H5N6
A/Eurasian_wigeon/Netherlands/1/2018_|_A_/_H5N6
A/jungle_crow/Hyogo/2803A002/2018_|_A_/_H5N6
A/jungle_crow/Hyogo/2803E022/2018_|_A_/_H5N6
A/jungle_crow/Hyogo/2803E011/2018_|_A_/_H5N6
Clade 2.3.4.4b
A/duck/Jiangxi/04.01_NCDZT0304-O/2015(Mixed)_|_A_/_H5N6
A/duck/Hunan/12.17_YYGK0057/2014(H5N6)_|_A_/_H5N6
A/muscovy_duck/Quang_Ninh/4c111/2013_|_A_/_H5N6
A/duck/Hunan/12.07_YYGK032-P/2013(H5N6)_|_A_/_H5N6
A/duck/Jiangsu/WX156/2013_|_A_/_H5N6
A/duck/Wenzhou/YHQL22/2014_|_A_/_H5N6
A/duck/Hunan/02.26_YYFQH290-P/2014(H5N6)_|_A_/_H5N6
A/environment/Zhenjiang/C13/2013_|_A_/_H5N6
A/chicken/Shenzhen/1845/2013_|_A_/_H5N6
A/duck/Zhejiang/6D2/2013_|_A_/_H5N6
Clade 2.3.4.4d
A/Duck/Guangdong/S3073/2010_|_A_/_H6N6
A/Duck/Guangdong/S3225/2010_|_A_/_H6N6
A/Duck/Guangdong/S3468/2010_|_A_/_H6N6
A/duck/Vietnam/LBM360c1-4-1/2013_|_A_/_H5N6
A/duck/Fujian/3076/2007_|_A_/_H6N6
A/duck/Hubei/03.06__WHWTZ0049-P/2015(Mixed)_|_A_/_H5N6
A/duck/Hubei/ZYSYG18/2015_|_A_/_H5N6
Clade 2.3.4.4a
A/duck/Fujian/4652/2007_|_A_/_H6N6
A/duck/Fujian/11418/2006_|_A_/_H6N6
A/duck/Fujian/12035/2005_|_A_/_H6N6
2002
2004
2006
2008
2010
2012
2014
2016
2018
2020

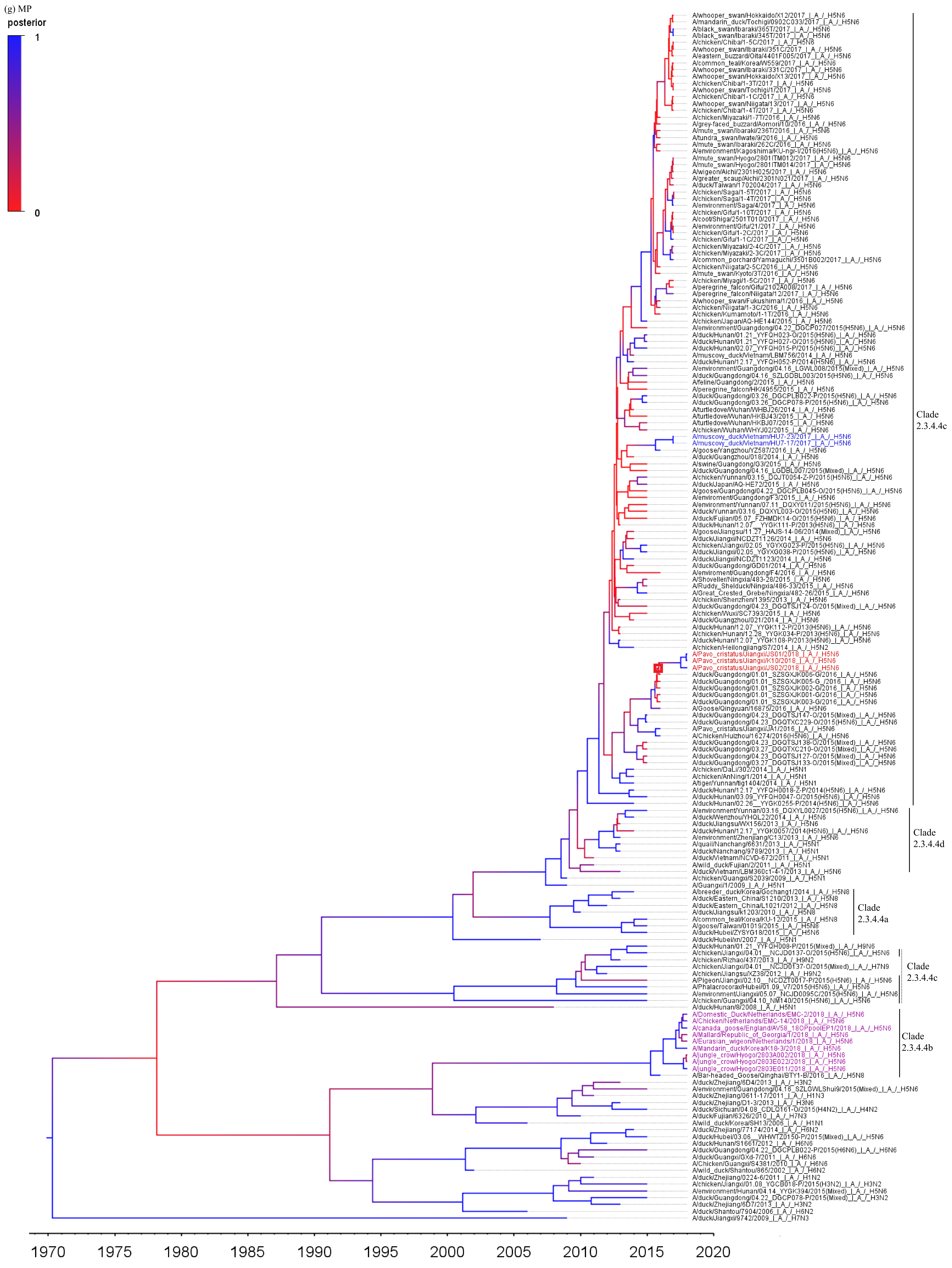
(g) MP
posterior
1
0
A/whooper_swan/Hokkaido/X12/2017_|_A_/_H5N6
A/mandarin_duck/Tochigi/0902C033/2017_|_A_/_H5N6
A/black_swan/Ibaraki/365T/2017_|_A_/_H5N6
A/black_swan/Ibaraki/345T/2017_|_A_/_H5N6
A/chicken/Chiba/1-5C/2017_|_A_/_H5N6
A/whooper_swan/Ibaraki/351C/2017_|_A_/_H5N6
A/eastern_buzzard/Oita/4401F005/2017_|_A_/_H5N6
A/common_teal/Korea/W559/2017_|_A_/_H5N6
A/whooper_swan/Ibaraki/331C/2017_|_A_/_H5N6
A/whooper_swan/Hokkaido/X13/2017_|_A_/_H5N6
A/chicken/Chiba/1-3T/2017_|_A_/_H5N6
A/whooper_swan/Tochigi/1/2017_|_A_/_H5N6
A/chicken/Chiba/1-1C/2017_|_A_/_H5N6
A/whooper_swan/Niigata/13/2017_|_A_/_H5N6
A/chicken/Chiba/1-4T/2017_|_A_/_H5N6
A/chicken/Miyazaki/1-7T/2016_|_A_/_H5N6
A/grey-faced_buzzard/Aomori/10/2016_|_A_/_H5N6
A/mute_swan/Ibaraki/236T/2016_|_A_/_H5N6
A/tundra_swan/Iwate/9/2016_|_A_/_H5N6
A/mute_swan/Ibaraki/262C/2016_|_A_/_H5N6
A/environment/Kagoshima/KU-ngr-I/2016(H5N6)_|_A_/_H5N6
A/mute_swan/Hyogo/2801ITM012/2017_|_A_/_H5N6
A/mute_swan/Hyogo/2801ITM014/2017_|_A_/_H5N6
A/wigeon/Aichi/2301H025/2017_|_A_/_H5N6
A/greater_scaup/Aichi/2301N021/2017_|_A_/_H5N6
A/duck/Taiwan/1702004/2017_|_A_/_H5N6
A/chicken/Saga/1-5T/2017_|_A_/_H5N6
A/chicken/Saga/1-4T/2017_|_A_/_H5N6
A/environment/Saga/4/2017_|_A_/_H5N6
A/chicken/Gifu/1-10T/2017_|_A_/_H5N6
A/coot/Shiga/2501T010/2017_|_A_/_H5N6
A/environment/Gifu/21/2017_|_A_/_H5N6
A/chicken/Gifu/1-2C/2017_|_A_/_H5N6
A/chicken/Gifu/1-1C/2017_|_A_/_H5N6
A/chicken/Miyazaki/2-4C/2017_|_A_/_H5N6
A/chicken/Miyazaki/2-3C/2017_|_A_/_H5N6
A/common_porchard/Yamaguchi/3501B002/2017_|_A_/_H5N6
A/chicken/Niigata/2-5C/2016_|_A_/_H5N6
A/mute_swan/Kyoto/3T/2016_|_A_/_H5N6
A/chicken/Miyagi/1-5C/2017_|_A_/_H5N6
A/peregrine_falcon/Gifu/2102A008/2017_|_A_/_H5N6
A/peregrine_falcon/Niigata/12/2017_|_A_/_H5N6
A/whooper_swan/Fukushima/1/2016_|_A_/_H5N6
A/chicken/Niigata/1-3C/2016_|_A_/_H5N6
A/chicken/Kumamoto/1-1T/2016_|_A_/_H5N6
A/chicken/Japan/AQ-HE144/2015_|_A_/_H5N6
A/environment/Guangdong/04.22_DGCP027/2015(H5N6)_|_A_/_H5N6
A/duck/Hunan/01.21_YYFQH023-O/2015(H5N6)_|_A_/_H5N6
A/duck/Hunan/01.21_YYFQH027-O/2015(H5N6)_|_A_/_H5N6
A/duck/Hunan/02.07_YYFQH015-P/2015(H5N6)_|_A_/_H5N6
A/muscovy_duck/Vietnam/LBM756/2014_|_A_/_H5N6
A/duck/Hunan/12.17_YYFQH052-P/2014(H5N6)_|_A_/_H5N6
A/environment/Guangdong/04.16_LGWL008/2015(Mixed)_|_A_/_H5N6
A/duck/Guangdong/04.16_SZLGDBL003/2015(H5N6)_|_A_/_H5N6
A/feline/Guangdong/2/2015_|_A_/_H5N6
A/peregrine_falcon/HK/4955/2015_|_A_/_H5N6
A/duck/Guangdong/03.26_DGCPLB022-P/2015(H5N6)_|_A_/_H5N6
A/duck/Guangdong/03.26_DGCP078-P/2015(H5N6)_|_A_/_H5N6
A/turtledove/Wuhan/WHBJ26/2014_|_A_/_H5N6
A/turtledove/Wuhan/HKBJ43/2015_|_A_/_H5N6
A/turtledove/Wuhan/HKBJ07/2015_|_A_/_H5N6
A/chicken/Wuhan/WHYJ02/2015_|_A_/_H5N6
A/muscovy_duck/Vietnam/HU7-23/2017_|_A_/_H5N6
A/muscovy_duck/Vietnam/HU7-17/2017_|_A_/_H5N6
A/goose/Yangzhou/YZ587/2016_|_A_/_H5N6
A/duck/Guangzhou/018/2014_|_A_/_H5N6
A/swine/Guangdong/G3/2015_|_A_/_H5N6
A/duck/Guangdong/04.16_LGDBL007/2015(Mixed)_|_A_/_H5N6
A/chicken/Yunnan/03.15_DQJT0054-Z-P/2015(H5N6)_|_A_/_H5N6
A/duck/Japan/AQ-HE72/2015_|_A_/_H5N6
A/goose/Guangdong/04.22_DGCPLB045-O/2015(H5N6)_|_A_/_H5N6
A/enviroment/Guangdong/F3/2015_|_A_/_H5N6
A/environment/Yunnan/07.11_DQXY011/2015(H5N6)_|_A_/_H5N6
A/duck/Yunnan/03.16_DQXYL003-O/2015(H5N6)_|_A_/_H5N6
A/duck/Fujian/05.07_FZHMDK14-O/2015(H5N6)_|_A_/_H5N6
A/duck/Hunan/12.07__YYGK111-P/2013(H5N6)_|_A_/_H5N6
A/goose/Jiangsu/11.27_HAJS-14-06/2014(Mixed)_|_A_/_H5N6
A/duck/Jiangxi/NCDZT1126/2014_|_A_/_H5N6
A/chicken/Jiangxi/02.05_YGYXG023-P/2015(H5N6)_|_A_/_H5N6
A/duck/Jiangxi/02.05_YGYXG038-P/2015(H5N6)_|_A_/_H5N6
A/duck/Jiangxi/NCDZT1123/2014_|_A_/_H5N6
A/duck/Guangdong/GD01/2014_|_A_/_H5N6
A/enviroment/Guangdong/F4/2016_|_A_/_H5N6
A/Shoveller/Ningxia/483-28/2015_|_A_/_H5N6
A/Ruddy_Shelduck/Ningxia/486-33/2015_|_A_/_H5N6
A/Great_Crested_Grebe/Ningxia/482-26/2015_|_A_/_H5N6
A/chicken/Shenzhen/1395/2013_|_A_/_H5N6
A/duck/Guangdong/04.23_DGQTSJ124-O/2015(Mixed)_|_A_/_H5N6
A/chicken/Wuxi/SC7393/2015_|_A_/_H5N6
A/duck/Guangzhou/021/2014_|_A_/_H5N6
A/duck/Hunan/12.07_YYGK112-P/2013(H5N6)_|_A_/_H5N6
A/chicken/Hunan/12.28_YYGK034-P/2013(H5N6)_|_A_/_H5N6
A/duck/Hunan/12.07_YYGK108-P/2013(H5N6)_|_A_/_H5N6
A/chicken/Heilongjiang/S7/2014_|_A_/_H5N2
A/Pavo_cristatus/Jiangxi/JS01/2018_|_A_/_H5N6
A/Pavo_cristatus/Jiangxi/K10/2018_|_A_/_H5N6
A/Pavo_cristatus/Jiangxi/JS02/2018_|_A_/_H5N6
A/duck/Guangdong/01.01_SZSGXJK006-G/2016_|_A_/_H5N6
A/duck/Guangdong/01.01_SZSGXJK005-G_/2016_|_A_/_H5N6
A/duck/Guangdong/01.01_SZSGXJK002-G/2016_|_A_/_H5N6
A/duck/Guangdong/01.01_SZSGXJK001-G/2016_|_A_/_H5N6
A/duck/Guangdong/01.01_SZSGXJK003-G/2016_|_A_/_H5N6
A/Goose/Qingyuan/16875/2016_|_A_/_H5N6
A/duck/Guangdong/04.23_DGQTSJ147-O/2015(Mixed)_|_A_/_H5N6
A/duck/Guangdong/04.23_DGQTXC229-O/2015(H5N6)_|_A_/_H5N6
A/Pavo_cristatus/Jiangxi/JA1/2016_|_A_/_H5N6
A/Chicken/Huizhou/16274/2016(H5N6)_|_A_/_H5N6
A/duck/Guangdong/04.23_DGQTSJ138-O/2015(Mixed)_|_A_/_H5N6
A/duck/Guangdong/03.27_DGQTXC210-O/2015(Mixed)_|_A_/_H5N6
A/duck/Guangdong/04.23_DGQTSJ127-O/2015(Mixed)_|_A_/_H5N6
A/duck/Guangdong/03.27_DGQTSJ133-O/2015(Mixed)_|_A_/_H5N6
A/chicken/DaLi/302/2014_|_A_/_H5N1
A/chicken/AnNing/1/2014_|_A_/_H5N1
A/tiger/Yunnan/tig1404/2014_|_A_/_H5N1
A/duck/Hunan/12.17_YYFQH0018-Z-P/2014(H5N6)_|_A_/_H5N6
A/duck/Hunan/03.09_YYFQH0047-O/2015(H5N6)_|_A_/_H5N6
A/duck/Hunan/02.26__YYGK0255-P/2014(H5N6)_|_A_/_H5N6
Clade 2.3.4.4c
A/environment/Yunnan/03.16_DQXYL0027/2015(H5N6)_|_A_/_H5N6
A/duck/Wenzhou/YHQL22/2014_|_A_/_H5N6
A/duck/Jiangsu/WX156/2013_|_A_/_H5N6
A/duck/Hunan/12.17_YYGK0057/2014(H5N6)_|_A_/_H5N6
A/environment/Zhenjiang/C13/2013_|_A_/_H5N6
A/quail/Nanchang/6631/2013_|_A_/_H5N1
A/duck/Nanchang/9789/2013_|_A_/_H5N1
A/duck/Vietnam/NCVD-672/2011_|_A_/_H5N1
A/wild_duck/Fujian/2/2011_|_A_/_H5N1
A/duck/Vietnam/LBM360c1-4-1/2013_|_A_/_H5N6
Clade 2.3.4.4d
A/chicken/Guangxi/S2039/2009_|_A_/_H5N1
A/Guangxi/1/2009_|_A_/_H5N1
A/breeder_duck/Korea/Gochang1/2014_|_A_/_H5N8
A/duck/Eastern_China/S1210/2013_|_A_/_H5N8
A/duck/Eastern_China/L1021/2012_|_A_/_H5N8
A/duck/Jiangsu/k1203/2010_|_A_/_H5N8
A/common_teal/Korea/KU-12/2015_|_A_/_H5N8
A/goose/Taiwan/01019/2015_|_A_/_H5N8
A/duck/Hubei/ZYSYG18/2015_|_A_/_H5N6
Clade 2.3.4.4a
A/duck/Hubei/xn/2007_|_A_/_H5N1
A/duck/Hunan/01.21_YYFQH008-P/2015(Mixed)_|_A_/_H9N6
A/chicken/Jiangxi/04.01__NCJD0137-O/2015(H5N6)_|_A_/_H5N6
A/chicken/Rizhao/437/2013_|_A_/_H9N2
A/chicken/Jiangxi/04.01__NCJD0137-O/2015(Mixed)_|_A_/_H7N9
A/chicken/Jiangsu/XZ38/2012_|_A_/_H9N2
A/Pigeon/Jiangxi/02.10__NCDZT0017-P/2015(H5N6)_|_A_/_H5N6
A/Phalacrocorax/Hubei/01.09_V7/2015(H5N6)_|_A_/_H5N6
A/environment/Jiangxi/05.07_NCJD0095C/2015(H5N6)_|_A_/_H5N6
A/chicken/Guangxi/04.10_NM140/2015(H5N6)_|_A_/_H5N6
Clade 2.3.4.4c
A/duck/Hunan/8/2008_|_A_/_H5N1
A/Domestic_Duck/Netherlands/EMC-2/2018_|_A_/_H5N6
A/Chicken/Netherlands/EMC-14/2018_|_A_/_H5N6
A/canada_goose/England/AV58_18OPpoolEP1/2018_|_A_/_H5N6
A/Mallard/Republic_of_Georgia/1/2018_|_A_/_H5N6
A/Eurasian_wigeon/Netherlands/1/2018_|_A_/_H5N6
A/Mandarin_duck/Korea/K18-3/2018_|_A_/_H5N6
A/jungle_crow/Hyogo/2803A002/2018_|_A_/_H5N6
A/jungle_crow/Hyogo/2803E022/2018_|_A_/_H5N6
A/jungle_crow/Hyogo/2803E011/2018_|_A_/_H5N6
A/Bar-headed_Goose/Qinghai/BTY1-B/2016_|_A_/_H5N8
Clade 2.3.4.4b
A/duck/Zhejiang/6D4/2013_|_A_/_H3N2
A/environment/Guangdong/04.16_SZLGWLShui9/2015(Mixed)_|_A_/_H5N6
A/duck/Zhejiang/0611-17/2011_|_A_/_H1N3
A/duck/Zhejiang/D1-3/2013_|_A_/_H3N6
A/duck/Sichuan/04.08_CDLQ161-O/2015(H4N2)_|_A_/_H4N2
A/duck/Fujian/6326/2010_|_A_/_H7N3
A/wild_duck/Korea/SH13/2006_|_A_/_H1N1
A/duck/Zhejiang/77174/2014_|_A_/_H6N2
A/duck/Hubei/03.06__WHWTZ0150-P/2015(Mixed)_|_A_/_H5N6
A/duck/Hunan/S1661/2012_|_A_/_H6N6
A/duck/Guangdong/04.22_DGCPLB022-P/2015(H6N6)_|_A_/_H6N6
A/duck/Guangxi/GXd-7/2011_|_A_/_H6N6
A/Chicken/Guangxi/S4381/2010_|_A_/_H6N6
A/wild_duck/Shantou/865/2002_|_A_/_H6N2
A/duck/Zhejiang/0224-6/2011_|_A_/_H1N2
A/chicken/Jiangxi/01.08_YGCB018-P/2015(H3N2)_|_A_/_H3N2
A/environment/Hunan/04.14_YYGK394/2015(Mixed)_|_A_/_H5N6
A/duck/Guangdong/04.22_DGCP078-P/2015(Mixed)_|_A_/_H3N2
A/duck/Zhejiang/6D7/2013_|_A_/_H3N2
A/duck/Shantou/7904/2006_|_A_/_H6N2
A/duck/Jiangxi/9742/2009_|_A_/_H7N3
1970
1975
1980
1985
1990
1995
2000
2005
2010
2015
2020

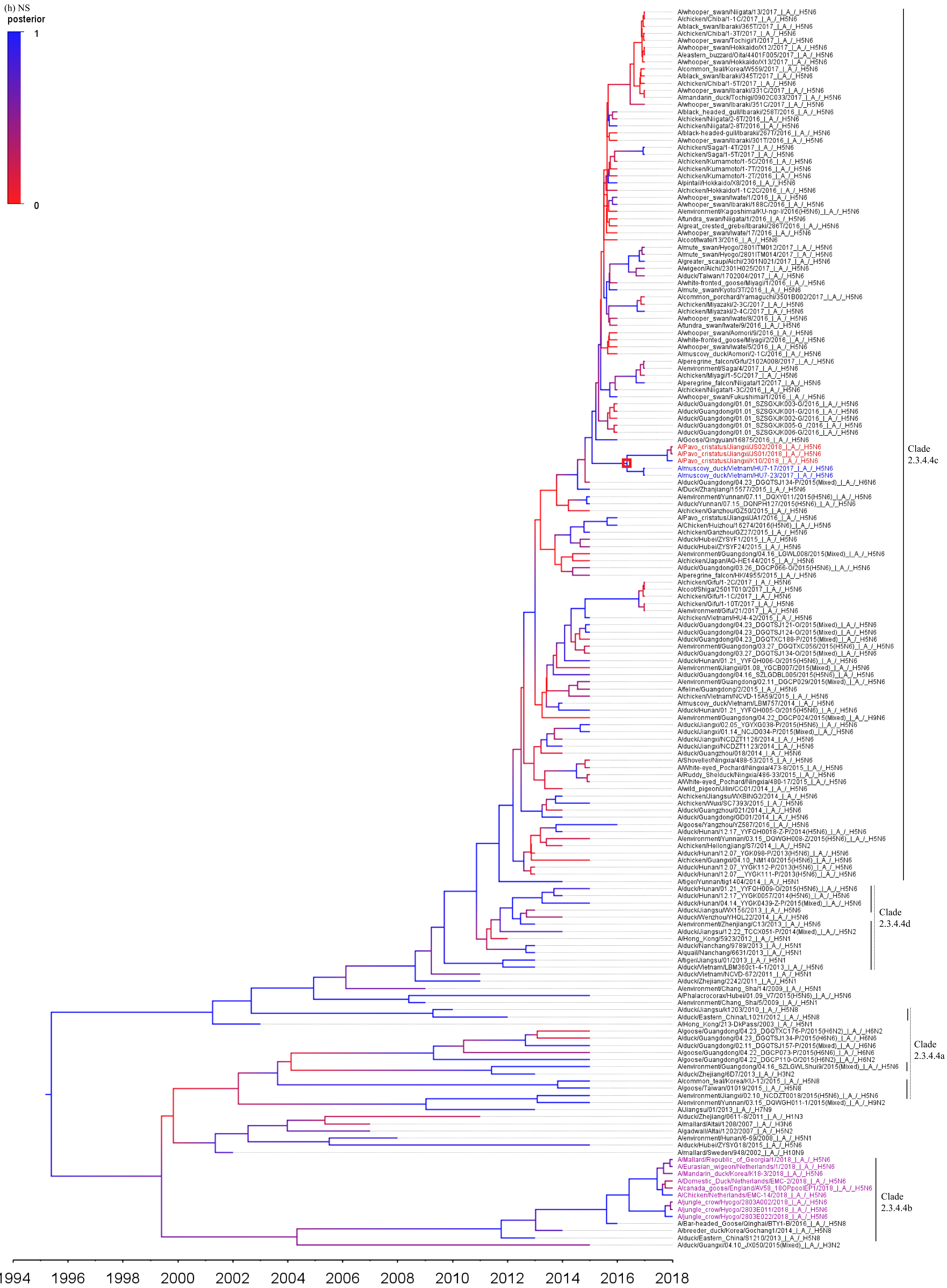

(h) NS
posterior
1
0
Clade 2.3.4.4c
Clade 2.3.4.4d
Clade 2.3.4.4a
Clade 2.3.4.4b
1994
1996
1998
2000
2002
2004
2006
2008
2010
2012
2014
2016
2018

Supplementary figure 3 MCC trees of influenza viruses internal genes generated by BEAST software (v1.8.4). Red squares represents the most recent common ancestor of the JS01, JS02, and K10 AIVs. The JS01, JS02, and K10 AIVs were marked by red text, the Hu7-17 and Hu7-23 AIVs were marked by blue text, and H5N6 AIVs isolated in 2018 were marked in purple text.
