# Supplementary material for: Highly pathogenic H5N6 avian influenza virus outbreak in Pavo cristatus in Jiangxi Province, China
Source: Emerg Microbes Infect. 2019 Mar 11;8(1):377–80. doi: 10.1080/22221751.2019.1586411 (PMC6455133; doi:10.1080/22221751.2019.1586411)
Supplement: Supplemental Material [file TEMI_A_1586411_SM9500.zip › Supplementary Material/Supplementary_materials.pdf]

Supplementary materials

Highly Pathogenic H5N6 Avian Influenza Virus Outbreak in *Pavo Cristatus* in Jiangxi Province, China

Meng Li[1], Shengyong Feng[1,2], Sanfu Lv[3], Jing Luo[1], Jianli Guo[3], Jianhua Sun[3], Hongxuan He[1,†]

1 National Research Center for Wildlife Borne Diseases, Institute of Zoology, Chinese Academy of Sciences, Beijing, PR China, 100101

2 College of Life Sciences, University of the Chinese Academy of Sciences, Beijing, PR China, 101407

3 Center of Animal Disease Prevention and Control of Tong Zhou District, Beijing, PR China, 100149

† Corresponding author; Email: hehx@ioz.ac.cn

Methods and materials

Ethics statement

Animal studies were performed in strict accordance with the Guidelines for the Care and Use of Animals in Research, issued by the Institute of Zoology, Chinese Academy of Sciences. This study has been evaluated and approved by Animal Ethics Committee of Institute of Zoology, Chinese Academy of Sciences. All experiments were conducted in Biosafety Level 3 (BSL-3) facility.

Samples collection

Four dead peafowls were necropsied. Tissue samples, including brains, tracheas, hearts, lungs, livers, stomachs, intestine, spleen, and kidneys were collected sterilely.

Oropharyngeal swabs and cloacal swabs were collected from 25 peafowls and 7 geese. All the samples were stored and transported on the ice.

Histopathological examination

The tissue samples were fixed in 4% paraformaldehyde, embedded in paraffin, and cut into 5-μm sections. The sections were stained with hematoxylin and eosin (H&E).

RT-PCR

Total RNAs were extracted using TRIzol Reagent (Invitrogen, Carlsbad, CA). The reverse-transcriptase PCR (RT-PCR) was performed using Access RT-PCR System (Promega, Madison, WI). The primers used in this analysis were listed as follows: M gene forward primer, 5'-GACCAATCCTGTCACCTCTGA-3', M gene reverse primer, 5'- GTATATGAGGCCCATRCAACT-3'. The reaction master mix was prepared according to the manufacture's instruction, and the reaction was conducted at 48 °C for 45 min, followed by 40 cycles of amplification at 95 °C for 30 sec, 55 °C for 45 sec, and 68 °C for 1 min, and a final amplification at 68 °C for 3 min. The resultant RT-PCR amplification products were examined by agarose gel electrophoresis.

Virus isolation, amplification, and sequencing

The virus was isolated using 11-day old embryonated SPF chicken eggs (Merial Vital laboratory, Beijing, China). Allantoic fluids were harvested from those embryonated eggs when the embryo died 24-48 hours after inoculation. Full genomes of JS01, JS02, and K10 virus were amplified using Phusion® High-Fidelity DNA Polymerase (NEB, Ipsich, MA). In detail, Primers used for genome sequencing were listed as below:

Uni12: 5-AGCRAAAGCAGG-3';

UnIA-F: 5'-GAAGTTGGGGGGGAGCRAAAGCAGG-3',

UnIA-R: 5'-CCGCCGGGTTATTAGTAGAAACAAGG-3'.

The amplification was performed at 98 °C for 30s, followed by 35 cycles of amplification at 98 °C for 15 sec, 45 °C for 30 sec, and 72 °C for 7 min, and a final elongation step at 72 °C for 10 min. The resultant RT-PCR amplification products were examined by 1% agarose gel electrophoresis. The PCR product was extracted using E.Z.N.A.® Gel Extraction Kit (Omega, Georgia, USA), and ligated into pEASY-Blunt vectors (Transgen, Beijing, China). The positive vectors was selected and sequenced (BGI, Beijing, China).

Phylogenetic analysis

All sequences used in this program were downloaded from the GISAID and the GenBank database. In detail, Blast analysis was performed on the NCBI and the GISAID database with default parameters using each of the JS01, JS02, and K10 genome sequences as a query. The first 250 gene sequences in the Blast output were collected. The reference sequences used for the H5-subtype influenza virus classifications were chosen based on guidance from OIE and previously published studies [1, 2]. In addition, we added some virus strains to each dataset to ensure the consistence of each MCC tree. Multiple sequence alignment were performed using the Influenza Research Database (IRD) [3].

To ensure the quality of the Bayesian analysis, we removed some virus strains from the dataset based on previously reported works [4]. Briefly, we constructed initial

trees using RAxML [5], Phylip [6], or MEGA5 to display the topological structure of each tree derived from each dataset. Based on the topological structure of the trees, we further deleted some sequences without impairing the topological structure of phylogenetic tree. We preserved all major lineages and all the most basal sequences in major clades. The nucleotide substitution model for each dataset was determined by jModeltest2.0. To estimate divergence times and rates of nucleotide substitutions, we used an uncorrelated relax-clock Bayesian Markov chain Monte Carlo method in BEAST v1.8.4 [7]. For each analysis, MCMC chains were run for 100 million or 200 million iterations and sampled every 10 thousand or 20 thousand iterations after a 10% burn-in to ensure an effective sample size was obtained for all posterior parameters, including the posterior, prior, likelihood, and meanRate (ESS>200). Maximum clade credibility (MCC) trees were obtained from the MCMC tree samples using TreeAnnotator v1.8.4. The resulting trees were edited and illustrated using FigTree (v1.4.3). More information about the datasets, nucleotide length, and best-fit models can be seen in Supplementary table 5.

Animal experiments

We inoculated 6-week-old chickens (n=10) (Merial Vital laboratory, Beijing, China) intravenously with 0.1 ml of diluted allantoic fluid (HA unit: 32). All of the chickens died due to the infection within 24 hours after inoculation. For mice infection experiment, groups of five 6- to 8-week-old BALB/c mice (Vital River Laboratory, Beijing, China) were anesthetized using CO_2 and then intranasally (i.n.) inoculated with 0.1 ml of serial 10-fold dilutions of infectious virus in PBS. The mice were

observed daily.

References

1. Beerens, N., et al., *Novel Highly Pathogenic Avian Influenza A(H5N6) Virus in the Netherlands, December 2017.* Emerg Infect Dis, 2018. **24**(4).
2. Bi, Y., et al., *Genesis, evolution and prevalence of H5N6 avian influenza viruses in China.* Cell host & microbe, 2016. **20**(6): p. 810-821.
3. Squires, R.B., et al., *Influenza research database: an integrated bioinformatics resource for influenza research and surveillance.* Influenza and other respiratory viruses, 2012. **6**(6): p. 404-416.
4. Worobey, M., G.-Z. Han, and A. Rambaut, *A synchronized global sweep of the internal genes of modern avian influenza virus.* Nature, 2014. **508**(7495): p. 254.
5. Stamatakis, A., *RAxML version 8: a tool for phylogenetic analysis and post-analysis of large phylogenies.* Bioinformatics, 2014. **30**(9): p. 1312-3.
6. Retief, J.D., *Phylogenetic analysis using PHYLIP.* Methods Mol Biol, 2000. **132**: p. 243-58.
7. Drummond, A.J. and R.R. Bouckaert, *Bayesian Evolutionary Analysis with BEAST*. 2015: Cambridge University Press.
