# Supplementary material for: Highly pathogenic H5N6 avian influenza virus outbreak in Pavo cristatus in Jiangxi Province, China
Source: Emerg Microbes Infect. 2019 Mar 11;8(1):377–80. doi: 10.1080/22221751.2019.1586411 (PMC6455133; doi:10.1080/22221751.2019.1586411)
Supplement: Supplemental Material [file TEMI_A_1586411_SM9500.zip › Supplementary Material/Supplementary_Table_1_to_Supplementary_Table_5.pdf]

**Supplementary table 1** Blast analysis of genome sequences of these isolated viruses.

| Genes | Virus Strains | Identity (%) |
|---|---|---|
| PB2 | A/Quail/Guangxi/GX-2/2017 (H5N6) | 98.50% |
| PB1 | A/Environment/Guangxi/42586/2015 (H5N6) | 98.59% |
| PA | A/Ostrich/Guangxi/GX-1/2017 (H5N6) | 98.84% |
| HA | A/duck/Guangdong/01.01 SZSGXJK005-G /2016 (H5N6) | 98.30% |
| NP | A/duck/Guangdong/01.01 SZSGXJK005-G /2016 (H5N6) | 99.00% |
| NA | A/duck/Guangdong/01.01 SZSGXJK007-G/2016 (H5N6) | 98.41% |
| M | A/duck/Guangdong/03.27 DGQTSJ133-O/2015 (Mixed) | 99.00% |
| NS | A/duck/Guangdong/01.01 SZSGXJK006-G/2016 (H5N6) | 98.40% |

**Supplementary table 2** The reference ancestor strains and the estimate divergence time of each gene segment from its tMRCA.

| Genes | Reference ancestor strains | Estimated divergence time | 95% HPD | |
|---|---|---|---|---|
| | | | Upper | Lower |
| PB2 | A/duck/Hunan/04.22 LDDX037-P/2015 (H5N6)<br>A/duck/Guangdong/04.23 DGQTXC229-O/2015 (H5N6) | Jun 2014 | Feb 2014 | Sep 2014 |
| PB1 | A/Environment/Guangxi/44389/2015 (H5N6)<br>A/chicken/Japan/AQ-HE144/2015 (H5N6) | May 2014 | Apr 2013 | Dec 2014 |
| PA | A/Goose/Qingyuan/16875/2016 (H5N6) | Aug 2015 | May 2015 | Nov 2015 |
| HA | A/great egret/Hong Kong/00032/2016 (H5N6) | Sep 2015 | May 2015 | Dec 2015 |
| NP | A/Goose/Qingyuan/16875/2016 (H5N6) | Sep 2015 | May 2015 | Oct 2015 |
| NA | A/great egret/Hong Kong/00032/2016 (H5N6) | Sep 2015 | Jul 2015 | Dec 2015 |
| M | A/duck/Guangdong/01.01 SZSGXJK006-G/2016 (H5N6) | Dec 2015 | Sep 2015 | Feb 2016 |
| NS | A/Muscovy duck/Vietnam/HU7-17/2017 (H5N6) | Jun 2016 | Aug 2015 | Dec 2016 |

**Supplementary table 3** Estimated evolutionary rates of each gene segments of H5N6 viruses by Bayesian analysis.

| **Genes** | **Substitution rate and 95% HPD ($10^{-3}$ substitution/site/year)** | | |
|---|---|---|---|
| | **Mean** | **Lower** | **Upper** |
| PA | 3.55 | 2.51 | 4.73 |
| PB1 | 3.08 | 2.39 | 3.68 |
| PB2 | 2.65 | 2.05 | 3.24 |
| HA | 4.24 | 3.14 | 5.32 |
| NP | 2.50 | 1.81 | 3.19 |
| NA | 4.40 | 3.47 | 5.25 |
| M | 2.09 | 1.41 | 2.71 |
| NS | 3.66 | 2.76 | 4.52 |

**Supplementary table 4** Molecular characteristics of isolated virus were analyzed using Influenza Research Database (IRD, https://www.fludb.org/)

| Viruses[a] | H5 clade | HA[b] | | | NA | NS1 | M2 | | | | | PB2 | | | | PB1 | | | PA | |
|---|---|---|---|---|---|---|---|---|---|---|---|---|---|---|---|---|---|---|---|---|
| | | Cleavage site | RBS[c] | | Stalk deletion | 5-aa deletion | Drug-resistance associated aa | | | | | Air-borne transmission | | | | aa residues | | PB1-F2 truncation | aa residues | |
| | | | 222 | 224 | 58 - 68 | 80 - 84 | 26 | 27 | 30 | 31 | 34 | 271 | 591 | 627 | 701 | 99 | 368 | | 409 | 672 |
| JS01 | 2.3.4.4 | RERRRKR | Q | G | Yes | Yes | L | V | A | S | G | T | Q | E | D | H | I | 1-39 | S | L |
| JS02 | 2.3.4.4 | RERRRKR | Q | G | Yes | Yes | L | V | A | S | G | T | Q | E | D | H | I | 1-39 | S | L |
| K10 | 2.3.4.4 | RERRRKR | Q | G | Yes | Yes | L | V | A | S | G | T | Q | E | D | H | I | 1-39 | S | L |
| CNIC-21099[d] | 2.3.4.4 | REKRRKR | Q | G | Yes | No | L | V | A | S | G | T | Q | E | D | H | I | 12-90 | S | L |
| IDCDC-RG43A[d] | 2.3.4.4 | RERRRKR | Q | G | - | No | L | V | A | N | G | T | Q | E | D | H | I | 1-39 | S | F |

[a] Abbreviations: CNIC-21099 represents A/Fujian-Sanyuan/21099/2017 (H5N6), IDCDC-RG43A represents A/gyrfalcon/Washington/41088-6/2014 (H5N8).

[b] H5 numbering

[c] RBS: Receptor binding site

[d] Reference viruses were selected based on candidate vaccine viruses recommended by CDC or CCDC

**Supplementary table 5** Dataset size, nucleotide length, best-fit model, and MCMC chain length for each gene segment.

| Datasets | Sequence numbers | Nucleotide length (nt) | Substitution models[a] | Relax-clock models[b] | Tree models | MCMC chain length[c] |
|---|---|---|---|---|---|---|
| PB2 | 180 | 2280 | GTR+G+I | UCED | Bayesian SkyGrid | 200 |
| PB1 | 193 | 2274 | GTR+G+I | UCED | Bayesian SkyGrid | 200 |
| PA | 192 | 2151 | GTR+G+I | UCED | Bayesian SkyGrid | 200 |
| HA | 183 | 1710 | GTR+G | UCED | Bayesian SkyGrid | 200 |
| NP | 164 | 1496 | GTR+G | UCED | Exponential Growth | 200 |
| NA | 183 | 1446 | GTR+G+I | UCED | Bayesian SkyGrid | 200 |
| M | 178 | 981 | GTR+G+I | UCED | Bayesian SkyGrid | 100 |
| NS | 174 | 837 | GTR+G+I | UCED | Bayesian SkyGrid | 100 |

[a] The nucleotide substitution models were determined by jModeltest2.

[b] UCED represents uncorrelated exponential clock model;

[c] The unit was million.
